# RU.521 mitigates subarachnoid hemorrhage-induced brain injury via regulating microglial polarization and neuroinflammation mediated by the cGAS/STING/NF-κB pathway

**DOI:** 10.1186/s12964-023-01274-2

**Published:** 2023-09-28

**Authors:** Jiang Shao, Yuxiao Meng, Kaikun Yuan, Qiaowei Wu, Shiyi Zhu, Yuchen Li, Pei Wu, Jiaolin Zheng, Huaizhang Shi

**Affiliations:** 1 Department of Neurosurgery, the First Affiliated Hospital of Harbin Medical University, Youzheng Street 23#, Nangang District, Harbin, 150001 Heilongjiang Province China; 2 Department of Neurology, the Second Affiliated Hospital of Harbin Medical University, Xuefu Road 246#, Nangang District, Harbin, 150001 Heilongjiang Province China

**Keywords:** Subarachnoid hemorrhage, Early brain injury, cGAS, STING, NF-κB, Microglia, Neuroinflammation

## Abstract

**Background:**

The poor prognosis of subarachnoid hemorrhage (SAH) is often attributed to neuroinflammation. The cGAS-STING axis, a cytoplasmic pathway responsible for detecting dsDNA, plays a significant role in mediating neuroinflammation in neurological diseases. However, the effects of inhibiting cGAS with the selective small molecule inhibitor RU.521 on brain injury and the underlying mechanisms after SAH are still unclear.

**Methods:**

The expression and microglial localization of cGAS following SAH were investigated with western blot analysis and immunofluorescent double-staining, respectively. RU.521 was administered after SAH. 2’3’-cGAMP, a second messenger converted by activated cGAS, was used to activate cGAS-STING. The assessments were carried out by adopting various techniques including neurological function scores, brain water content, blood–brain barrier permeability, western blot analysis, TUNEL staining, Nissl staining, immunofluorescence, morphological analysis, Morris water maze test, Golgi staining, CCK8, flow cytometry in the in vivo and in vitro settings.

**Results:**

Following SAH, there was an observed increase in the expression levels of cGAS in rat brain tissue, with peak levels observed at 24 h post-SAH. RU.521 resulted in a reduction of brain water content and blood–brain barrier permeability, leading to an improvement in neurological deficits after SAH. RU.521 had beneficial effects on neuronal apoptosis and microglia activation, as well as improvements in microglial morphology. Additionally, RU.521 prompted a shift in microglial phenotype from M1 to M2. We also noted a decrease in the production of pro-inflammatory cytokines TNF-α, IL-1β, and IL-6, and an increase in the level of the anti-inflammatory cytokine IL-10. Finally, RU.521 treatment was associated with improvements in cognitive function and an increase in the number of dendritic spines in the hippocampus. The therapeutic effects were mediated by the cGAS/STING/NF-κB pathway and were found to be abolished by 2’3’-cGAMP. In vitro, RU.521 significantly reduced apoptosis and neuroinflammation.

**Conclusion:**

The study showed that SAH leads to neuroinflammation caused by microglial activation, which contributes to early brain injury. RU.521 improved neurological outcomes and reduced neuroinflammation by regulating microglial polarization through the cGAS/STING/NF-κB pathway in early brain injury after SAH. RU.521 may be a promising candidate for the treatment of neuroinflammatory pathology after SAH.

Video Abstract

**Supplementary Information:**

The online version contains supplementary material available at 10.1186/s12964-023-01274-2.

## Background

Spontaneous subarachnoid hemorrhage (SAH) is a severe subtype of stroke that occurs in about 5% of cases. It leads to the loss of many years of productive life span and is primarily caused by the rupture of an intracranial aneurysm in 85% of cases. Unfortunately, this often results in poor outcomes [1, 2]. Several processes are involved in the mechanisms underlying brain injury after SAH, including neuroinflammation, oxidative stress injury, ferroptosis, mitophagy, and neuronal apoptosis [3–7]. In spite of significant progress in the treatment of SAH, patient prognosis remains unsatisfactory. Therefore, it is crucial to gain a deeper understanding of the underlying pathological mechanisms of SAH to develop novel therapeutic strategies for affected individuals.

Neuroinflammation is characterized by the activation of immune cells and the production of pro-inflammatory cytokines such as IL-1β, IL-6, IL-18, and tumor necrosis factor (TNF), chemokines including C–C motif chemokine ligand 1 (CCL1), CCL5, and C-X-C motif chemokine ligand 1 (CXCL1), small-molecule messengers like prostaglandins and nitric oxide (NO), and reactive oxygen species by innate immune cells within the central nervous system (CNS) [8, 9]. It is a fundamental response to maintaining CNS homeostasis. However, prolonged or excessive neuroinflammation can be harmful and may result in neuronal damage and neurological dysfunction [10–12].

Microglia, the resident immune cells of the brain, are important mediators of neuroinflammation [13, 14]. Morphologically, microglia in a state of homeostasis exhibit a highly ramified structure. However, when activated, microglia can assume polarized phenotypes, namely the classically-activated state (M1 phenotype) which is proinflammatory and marked by specific indicators such as CD16, CD86, and iNOS, or the alternatively-activated state (M2 phenotype) which is anti-inflammatory and marked by specific indicators such as CD206, CD163, and Arg-1 [15–17]. Previous studies have confirmed the extensive involvement of microglia in the occurrence and development of neuroinflammation in early brain injury [16]. Recent studies show that improving outcomes in neurological diseases, such as subarachnoid hemorrhage, can be achieved by modulating signaling pathways involved in microglial activation and neuroinflammation [5, 18, 19].

Proper compartmentalization of DNA within the nucleus and mitochondria is crucial for maintaining the integrity of eukaryotic cells. It has been widely recognized that foreign or mislocalized endogenous DNA can trigger innate immunity, highlighting the importance of maintaining proper DNA localization within the cell [20]. As a mammalian cytosolic DNA sensor, dimeric cyclic GMP-AMP synthase (cGAS) binds to DNA ligands and is activated in a length-dependent manner, forming ladder-like networks cooperatively [20]. Activated cGAS converts ATP and GTP to cyclic GMP-AMP (cGAMP) [21]. cGAMP is a second messenger that activates the Stimulator of Interferon Genes (STING) protein which is located in the Endoplasmic Reticulum (ER) [22]. STING, a protein involved in innate immune response, forms dimers and moves from the ER to perinuclear structures like the Golgi Apparatus. It then binds to TANK binding kinase 1 (TBK1) which phosphorylates it. The phosphorylated STING then interacts with the positively charged surfaces of interferon regulatory factor 3 (IRF3), leading to its phosphorylation and activation by TBK1. IRF3, a master regulator of Type I interferons, then translocates to the nucleus and activates the transcription of genes encoding interferons and other cytokines. NF-κB, a transcription factor, is also activated in response to inflammatory stimuli [23]. RU.521, a small molecule inhibitor of cGAS, has demonstrated activity and selectivity in cellular assays of cyclic GMP-AMP synthase-mediated signaling. Furthermore, it has been shown to reduce the constitutive expression of interferon in macrophages from a mouse model of Aicardi-Goutieres syndrome [24]. The use of RU.521 to inhibit the cGAS-STING signaling pathway has been found to effectively suppress microglial M1-polarization in the spinal cord, resulting in a reduction of neuropathic pain symptoms [19]. The exact effect and mechanism of RU.521 in the pathological process that occurs after SAH has not yet been fully understood.

Based on the evidence presented, the hypothesis was that the cGAS-STING pathway could contribute to microglial polarization and neuroinflammation after experimental subarachnoid hemorrhage. Pharmacological inhibition of the cGAS-STING signaling pathway with RU.521 could potentially mitigate subarachnoid hemorrhage-induced brain injury by regulating microglial polarization and neuroinflammation. To investigate the effects and mechanisms of RU.521 under SAH conditions, both in vivo and in vitro SAH models were utilized.

## Materials and methods

### Animal

Male Sprague–Dawley rats weighing between 280–320 g were obtained from the Animal Center of the First Affiliated Hospital of Harbin Medical University. The rats were housed in a room with a controlled temperature of 25–28 °C and appropriate humidity. Before modeling, all rats were given ad libitum access to food and water. The animal experiments conducted in this study adhered to the guidelines set forth by the National Institutes of Health (NIH) and were approved by the Institutional Animal Care and Use Committee of the First Affiliated Hospital of Harbin Medical University.

### Experimental design

All experiments were performed as shown in Supplementary Figure S1.

#### Experiment 1

To determine the endogenous expression of cGAS in the ipsilateral (left) basal temporal lobe cortex, Western blots were performed in the Sham group and in the SAH groups that were randomly divided into 6, 12, 24, and 72 h after SAH (*n* = 6 per group). To investigate the co-localization of cGAS with microglial Iba1, immunofluorescence staining was conducted at 24 h post-operation in both the Sham and SAH models (*n* = 6 per group).

#### Experiment 2

To evaluate the neurological outcome, brain edema, and blood–brain barrier disruption, a total of five groups of rats were randomly assigned (Sham, SAH + Vehicle (1% DMSO + corn oil), SAH + RU.521 (150 μg/kg), SAH + RU.521 (450 μg/kg), and SAH + RU.521 (1350 μg/kg). *n* = 12 per group). After analyzing the results obtained from each group 24 h after SAH, we determined that the appropriate dose for the following experiments would be 450 μg/kg RU.521.

#### Experiment 3

To assess the neuronal injury and the activation of microglia after SAH, 36 rats (*n* = 12 per group) were randomly assigned into 3 groups: Sham, SAH + Vehicle, and SAH + RU.521(450 μg/kg). To investigate neuronal damage after SAH, TUNEL and Nissl staining were conducted 24 h post-injury. Furthermore, western blots and immunofluorescence (Iba1) were performed at the same time point to assess activated microglia in each group. The methods used to analyze microglia morphology were previously reported [25].

#### Experiment 4

To investigate the specific role of RU.521 and related molecular mechanisms, rats were randomly divided into 5 groups: SAH, SAH + Vehicle, SAH + RU.521(450 μg/kg), SAH + PBS + RU.521(450 μg/kg), and SAH + 2’3’-cGAMP + RU.521 (450 μg/kg). Western blots, immunofluorescence, Morris water maze test, and Golgi staining were adopted for assessments.

#### Experiment 5

The HT22 hippocampal neurons and BV2 microglial cells of mice, obtained from Procell, China, were cultured in Dulbecco’s modified Eagle’s medium (DMEM) from Gibco, USA. The medium was supplemented with 10% fetal bovine serum (FBS) from HyClone, PA, USA and 1% penicillin–streptomycin from Gibco, USA. The cells were cultured in a 37 °C, 5% CO_2_ incubator. To replicate the SAH in vitro, BV2 cells were subjected to oxyhemoglobin (Oxy-Hb; Sigma–Aldrich, USA) at a concentration of 10 μM. Additionally, 1% DMSO was used as a control, as previously reported [5]. After exposing BV2 cells to Oxy-Hb for 1 h in the chamber, the medium was mixed with RU.521 for subsequent periods. Transwell plates (Corning, USA) were used for microglia-neuron cocultures at the indicated time. BV2 cells were placed in the upper chamber with fresh medium, while HT22 cells were seeded in the lower chamber of the plates for incubation at 37 °C and 5% CO_2_ in a humid environment until the following experiments.

### SAH model

Endovascular perforation was performed to induce experimental SAH in rats as previously described [5, 6]. Briefly, the external carotid artery (ECA) and internal carotid artery (ICA) were carefully exposed under satisfactory anesthetization using pentobarbital sodium. A sharp 4–0 nylon suture was inserted into the left ICA from the cut of the ECA stump until resistance was felt. The suture was advanced several times to puncture the artery, then immediately withdrawn. For the Sham group, all procedures were carried out except for the puncture of the vessel.

### SAH grading

The severity of SAH was assessed by two researchers using the SAH grading scale described previously [26]. In brief, after inducing SAH, the rats were anesthetized and sacrificed 24 h later. The ventral side of the brain was then divided into six parts (Supplementary Figure S2). The volume of blood clots in the basal surface was measured using a 0–3 score system (Supplementary Table S1) for each part. The rats were scored on six different metrics. Rats with an average score of less than 7 were excluded from further experiments.

### Drug administration

RU.521 (HY-114180, MedChemExpress) was dissolved in 1% DMSO + corn oil (DMSO, Sigma-Aldrich; corn oil, HY-Y1888. MedchemExpress) and three doses of RU.521 (150 μg/kg, 450 μg/kg, and 1350 μg/kg) were intranasally administrated 1 h after SAH. 2’3’-cGAMP(500 μg/kg, HY-100564, MedChemExpress) or vehicle (phosphate-buffered saline, PBS) was intranasally administrated 1 h before SAH. For inhibition of cGAS in vitro, BV2 cells were treated with 500 nM RU.521 after exposure to Oxy-Hb for 1 h. The same volume of 1% DMSO was used as the control treatment. The drug-delivery way and dosage mentioned above were based on previous research [19, 27, 28].

### Assessment of neurological outcomes

After 24 h of SAH, neurological outcomes were evaluated by two independent investigators using modified Garcia scores and beam balance tests. Additional information on the neurobehavior evaluation can be found in Supplementary Tables S2 and S3, as previously described [5, 6], the scores of three consecutive trials were averaged for analysis. Higher scores indicated better neurological function.

### Brain edema and blood–brain barrier disruption

Brain water content was assessed to determine brain edema by measuring the wet and dry weight of the rat brains 24 h after SAH induction. The brains were first weighed while wet after the removal of clotted blood and then dried in an oven at 100 °C for 24 h to obtain the dry weight. The percentage of brain water content was calculated using the formula [(wet weight—dry weight) / wet weight] × 100%.

The disruption of the BBB was evaluated with Evan’s Blue (Sigma, USA) extravasation 24 h after SAH, as previously reported [29]. To measure the severity of BBB disruption, Evan’s Blue (4%, 5 mL/kg) was injected into the left ventricle 1 h before sacrificing the animals. The brain tissues were then weighed after transcardial perfusion with normal saline and homogenized in 50% trichloroacetic acid (TCA). The homogenized brain tissues were centrifuged and the supernatant was collected. The supernatant was mixed with ethanol and TCA and then maintained overnight at 4 °C. The Evan's Blue concentration in the supernatant was determined by measuring the absorbance at 630 nm.

### Nissl staining

In this study, rats were sacrificed at specific time points after SAH and their brains were perfused with cold phosphate buffer solution (PBS) and 4% paraformaldehyde (PFA). The brains were then fixed in PFA solution at 4 °C for 24 h and transferred to a 30% sucrose solution until dehydration. The brain tissue was embedded and frozen in the Tissue-Tek O.C.T. compound (Sakura Finetek, USA) and cut into 10 μm or 20 μm frozen coronal sections using a Leica CM1950 cryostat for further analysis. To identify degenerative neurons, Nissl staining was performed using 5% cresyl violet (Beyotime, China) and observed under light microscopy (Lecia Microsystems, DMi8, Germany). Degenerative neurons were characterized by a shrunken cytoplasm and condensed staining. The mean number of surviving neurons was calculated to assess neuronal loss [6].

### Immunofluorescence and TUNEL staining

For immunofluorescence staining, brain sections and BV2 cells coverslips fixed with 4% paraformaldehyde solution were blocked with 5% bovine serum albumin (BSA) and 0.1% Triton X-100 for 1 h at room temperature. The sections and coverslips were then incubated overnight at 4 °C with the indicated primary antibodies (Supplementary Table S4). After being washed with phosphate-buffered saline (PBS), the sections were incubated with suitable secondary antibodies for 1 h at room temperature. To perform NeuN and TUNEL co-staining, the sections were initially stained with NeuN antibody overnight at 4 °C, followed by Apoptosis Detection Kit (Roche, USA) to detect apoptotic cell death as per the manufacturer’s protocol. To evaluate neuronal apoptosis, the number of TUNEL-positive neurons in the cortex was measured. The mean number of target cells in brain sections was measured by independent observers. Images were captured using a fluorescence microscope (Lecia Microsystems, DMi8, Germany).

### Microglia morphometric analysis

To quantify microglia morphology, images of Iba1 positive cells in a 20 μm brain section were analyzed using Image J software (Image J, NIH, USA). Specifically, the AnalyzeSkeleton (2D/3D) and FracLac plugins were utilized, as previously reported [25, 30, 31]. For the purpose of skeleton analysis, the fluorescence photomicrographs were first converted to grayscale to ensure optimal visualization of all positive staining. The brightness and contrast were adjusted as needed, and an Unsharp Mask filter was applied to enhance contrast. Salt-and-pepper noise was removed through a despeckle step, and the image was then converted to binary. The despeckle, close-, and remove outliers functions were applied before skeletonizing the image to run the AnalyzeSkeleton(2D/3D) plugin. Copy the data in the Results and Branch information outputs for further statistical analysis. To perform fractal analysis, convert the binary cell to an outline and use the FracLac plugin. Select BC (box counting) and set Num G to 4 in the Grid Design settings. Check the Metrics box under Graphics options to analyze the convex hull and bounding circle. Click on the Scan button and copy the desired data such as density, span ratio, circularity, fractal dimension, and lacunarity for further statistical analysis.

### Golgi staining

Golgi staining was performed with the FD Rapid GolgiStain Kit (FD Neurotechnologies, Columbia, SC, USA) according to the manufacturer’s protocol as previously reported [32]. Following their removal and a brief rinse in double distilled water, the brains were immersed in impregnation solutions (A/B) and left at room temperature for two weeks. Following this, they were stored in solution C for 72 h before being cut into 150 μm thick coronal sections using a cryostat (CM1950, Leica Microsystems, Bannockburn, Germany) and mounted on microscope slides coated with gelatin. Following a 10-min staining process, the sections were rinsed with double distilled water and dehydrated. This was followed by a xylene treatment and coverslipping with neutral balsam mounting medium. Finally, the sections were observed under a NIKON Eclipse ci microscope (NIKON, Japan) using an extended depth of focus (EDF) mode. In this study, we measured the density of dendritic spines in the hippocampus by examining the second or third dendritic terminal branches. Specifically, we analyzed the number of spines per 10 μm. An independent investigator was responsible for identifying, counting, and analyzing the dendritic spine density.

### Morris water maze test

The spatial learning and memory abilities were assessed using the Morris water maze test on days 22 to 28 after SAH, following previously established protocols [33, 34]. After a 22-day visible platform trial, rats were tasked with finding a submerged platform (10 cm in diameter) located 2 cm below the water level in a 180 cm diameter pool. This hidden platform trial was conducted three times per day for five consecutive days, with the animals allowed to remain on the platform for 10 s after finding it or being guided to it. This task was performed 23–27 days after SAH. During the probe trial, the animals were given 120 s to swim from a fixed starting position without a platform in the pool. We recorded various parameters such as escape latency, swim distance, number of times each animal crossed the original position of the platform, swimming speed, and swimming path for further analysis. The investigators who carried out the entire procedure were unaware of the group design of this study.

### Flow cytometry

To assess HT22 cell apoptosis, Flow cytometry and Annexin V-FITC Apoptosis Detection Kit (Beyotime, China) were used, as previously reported [4]. Data were acquired and analyzed by NovoCyte Express (ACEA, Biosciences, USA). Apoptosis rates were calculated with the following formula: [(AnnexinV^+^/PI^−^cells + AnnexinV^+^/PI^+^cells)/total cells] × 100%.

### Cell viability assay

The viability of HT22 cells was assessed using the Cell Counting Kit-8 (CCK-8) from Beyotime, China. Briefly, after coculture, 1000 HT22 cells were seeded in each well of a 96-well plate with 100 μL of working solution (10μL CCK-8 reagent + 90 μL DMEM) and incubated for 1.5 h. The absorbance of each well was measured at 450 nm wavelength using an ELx800 spectrophotometer from Biotek, USA. Each group had three replicate wells and the experiment was repeated three times.

### Western blot analysis

Western blot analysis was performed as described previously [5, 6]. Brain tissue or cell lysates were prepared using either RIPA lysis buffer or a nucleoprotein extraction kit from Beyotime (China) following the manufacturer's instructions. Equal amounts of protein were separated using 8–10% SDS-PAGE gel and then transferred onto a polyvinylidene fluoride (PVDF) membrane from Millipore (USA). After blocking with 5% skimmed milk or BSA for 1 h at room temperature, the membrane was incubated overnight at 4 °C with primary antibodies. Subsequently, appropriate HRP-conjugated secondary antibodies were added and incubated at room temperature for 1 h. Supplementary Table S4 lists the antibodies used. The membranes were then washed three times and detected using an enhanced chemiluminescence (ECL) reagent kit (NCM Biotech, China). The resulting images were analyzed using Image J software (NIH, USA).

### Statistical analysis

Statistical analysis was performed using GraphPad Prism 9.0 software (GraphPad Software, USA). The sample size was based on the previous reports [5, 6, 35]. All the data were presented as mean ± error of the mean (SEM). One-way analysis of variance (ANOVA) followed by Tukey’s post hoc test was performed for comparisons among multiple groups. The Kruskal–Wallis H test was used for multiple groups to test variables that were not normally distributed. In addition, two-way repeated measures ANOVA were applied to analyze the data of long-term neurological functions. A two-tailed *P* value < 0.05 was defined as statistically significant (*: *P* < 0.05, **: *P* < 0.01, ***: *P* < 0.001, and ****: *P* < 0.0001).

## Results

### Mortality rates and SAH grade

In this study, a total of 297 rats were utilized. Among them, 54 rats were assigned to the Sham group, while the remaining 243 rats were used to establish the SAH model. Out of the SAH group, 17 rats were excluded due to their SAH grade score being less than 7, and 52 rats died due to severe SAH or complications of anesthesia. The overall mortality rate of the SAH group was found to be 21.4% (52/243) (Supplementary Table S5). Supplementary Figure S2A displayed images of both sham and successful subarachnoid hemorrhage. The blood clots were primarily located around the circle of Willis and the ventral side of the brain stem following endovascular perforation. SAH grade scores did not exhibit any significant differences among the various SAH groups (Supplementary Figure S2B).

### Time course and microglial localization of cGAS

**Fig. 1:**
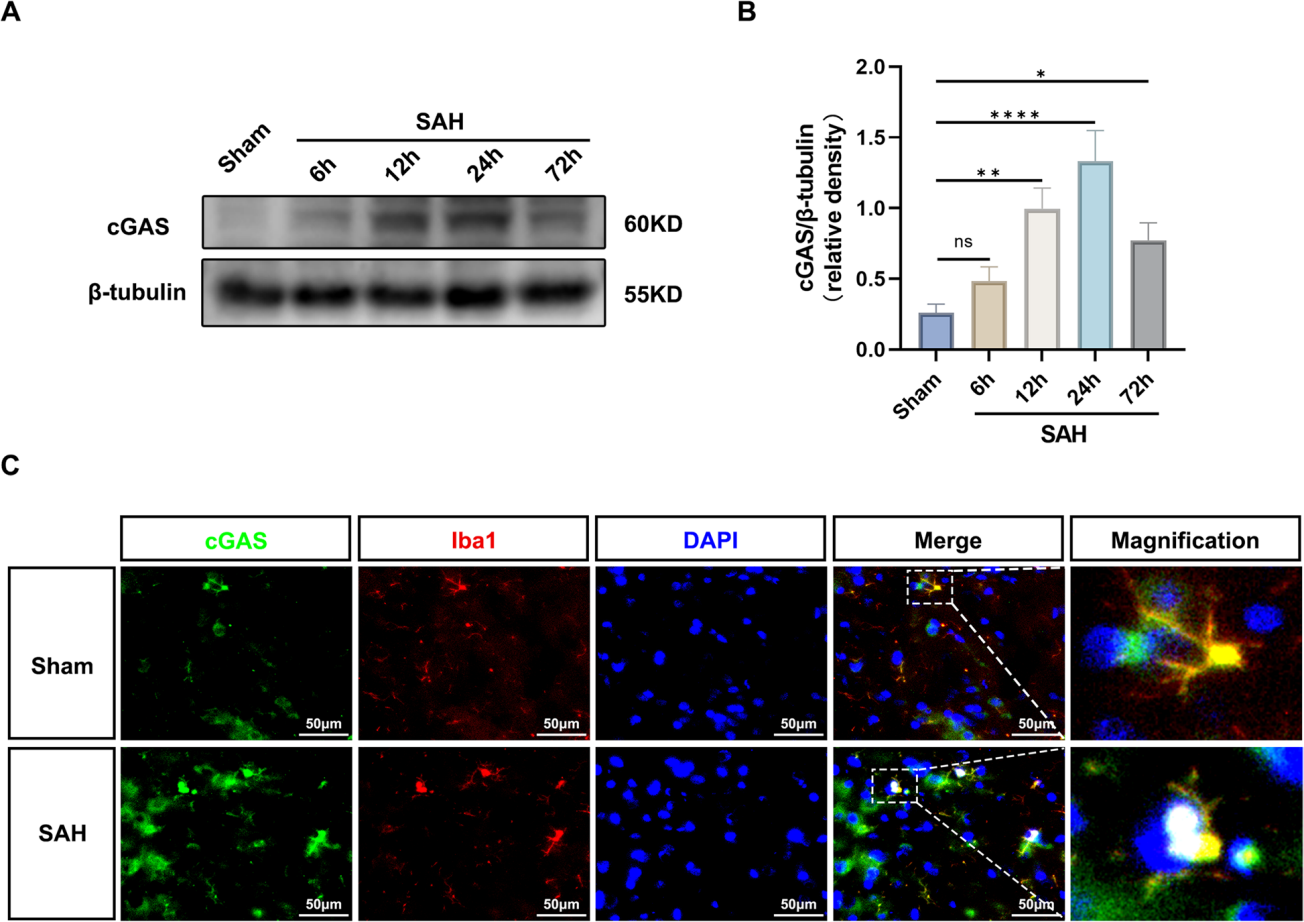
Expression changes of cGAS and microglial localization. **A**, **B** Representative Western blot bands of time course and quantitative analysis for cGAS. *n* = 6 per group. *: *P* < 0.05, **: *P* < 0.01, ***: *P* < 0.001, and ****: *P* < 0.0001. ns, not significant. **C** Representative images of double immunofluorescence staining for cGAS (green) with microglia (Iba1, red) at 24 h after SAH. Cell nuclei were counterstained with DAPI (blue). Scale bar = 50 μm. *n* = 6 per group

In this study, rats were randomly assigned to five groups, including a sham group and four subgroups with SAH at different time points (6 h, 12 h, 24 h, and 72 h post-SAH). The expression of cGAS in the left ventral cortex was analyzed by Western blots. The results showed that cGAS expression started to increase at 6 h after SAH, peaked at 24 h, and decreased at 72 h after SAH (Fig. 1A, B). The co-localization of cGAS and microglia in the ventral cortex after SAH was further confirmed through double immunofluorescence staining of cGAS and Iba1 (Fig. 1C).

### RU.521 improved the neurological score and reduced neuronal injury after SAH

**Fig. 2:**
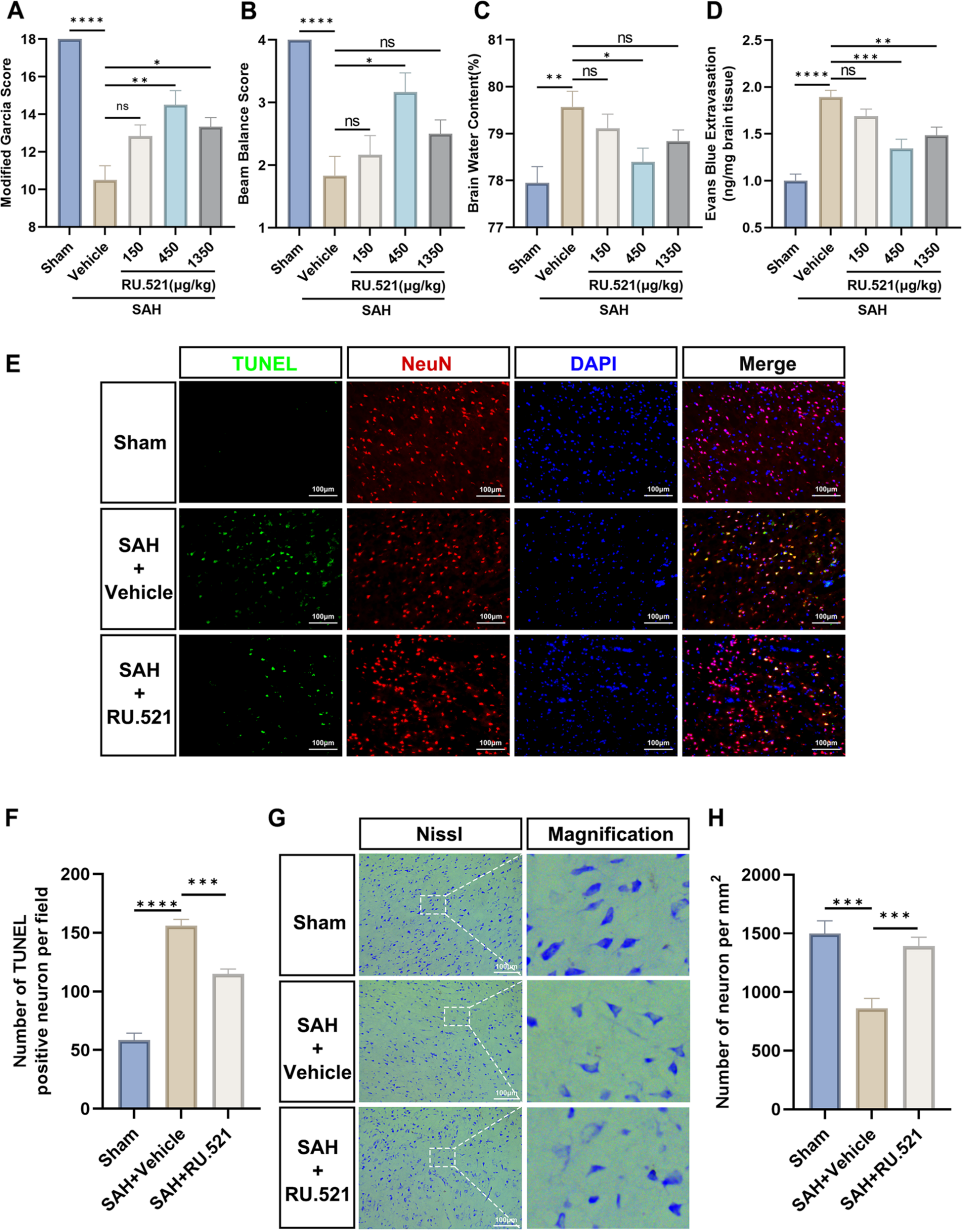
RU.521 was found to have a positive impact on neurological deficits, brain edema, BBB disruption, and neuronal damage 24 h after SAH. **A**, **B** The modified Garcia and beam balance scores of each group. *n* = 6 per group. **C** Quantification of brain water content at 24 h after SAH. *n* = 6 per group. **D** Quantification of Evans blue extravasation at 24 h after SAH. *n* = 6 per group. **E** Representative images of TUNEL staining (green) with neurons (NeuN, red). Scale bar = 100 μm. **F** Quantitative analysis of TUNEL-positive neuronal cells. *n* = 6 per group. **G** Representative images of Nissl staining. Scale bar = 100 μm. **H** Quantitative analysis of Nissl staining. *n* = 6 per group. *: *P* < 0.05, **: *P* < 0.01, ***: *P* < 0.001, and ****: *P* < 0.0001. ns, not significant

Neurological function was evaluated 24 h after SAH using the modified Garcia score and beam balance tests. The neurological function significantly deteriorated after SAH when compared with the Sham group (Fig. 2A, B). The medium dose (450 μg/kg) of RU.521 administered intranasally was found to alleviate the decline in both the modified Garcia score and beam balance tests (Fig. 2A, B). Consistently, the brain water content in the SAH + Vehicle group was significantly higher than the Sham group 24 h after SAH (Fig. 2C). Administration of RU.521 at a medium dose of 450 μg/kg resulted in significant alleviation of brain edema after SAH (Fig. 2C). Following SAH, there was a significant increase in the extravasation of Evan's blue. However, treatment with RU.521 was found to alleviate this increased extravasation (Fig. 2D). The subsequent studies for SAH in rats utilized the medium dose of RU.521, which was determined to be the optimal dose at 450 μg/kg.

Neuronal apoptosis was evaluated using TUNEL staining. The findings revealed a significant increase in the number of apoptotic neurons in the SAH + Vehicle group 24 h after SAH (Fig. 2E, F). However, the administration of RU.521 alleviated neuronal apoptosis (Fig. 2E, F).

Nissl staining was conducted to assess the neuron loss and shrinkage morphology in the cortex. The findings indicated an increase in neuron loss and shrinkage morphology in the SAH + Vehicle group (Fig. 2G, H). However, the administration of RU.521 exhibited a significant reduction in neuron loss (Fig. 2G, H).

### RU.521 blunted microglia activation and morphological changes 24 h after SAH

**Fig. 3:**
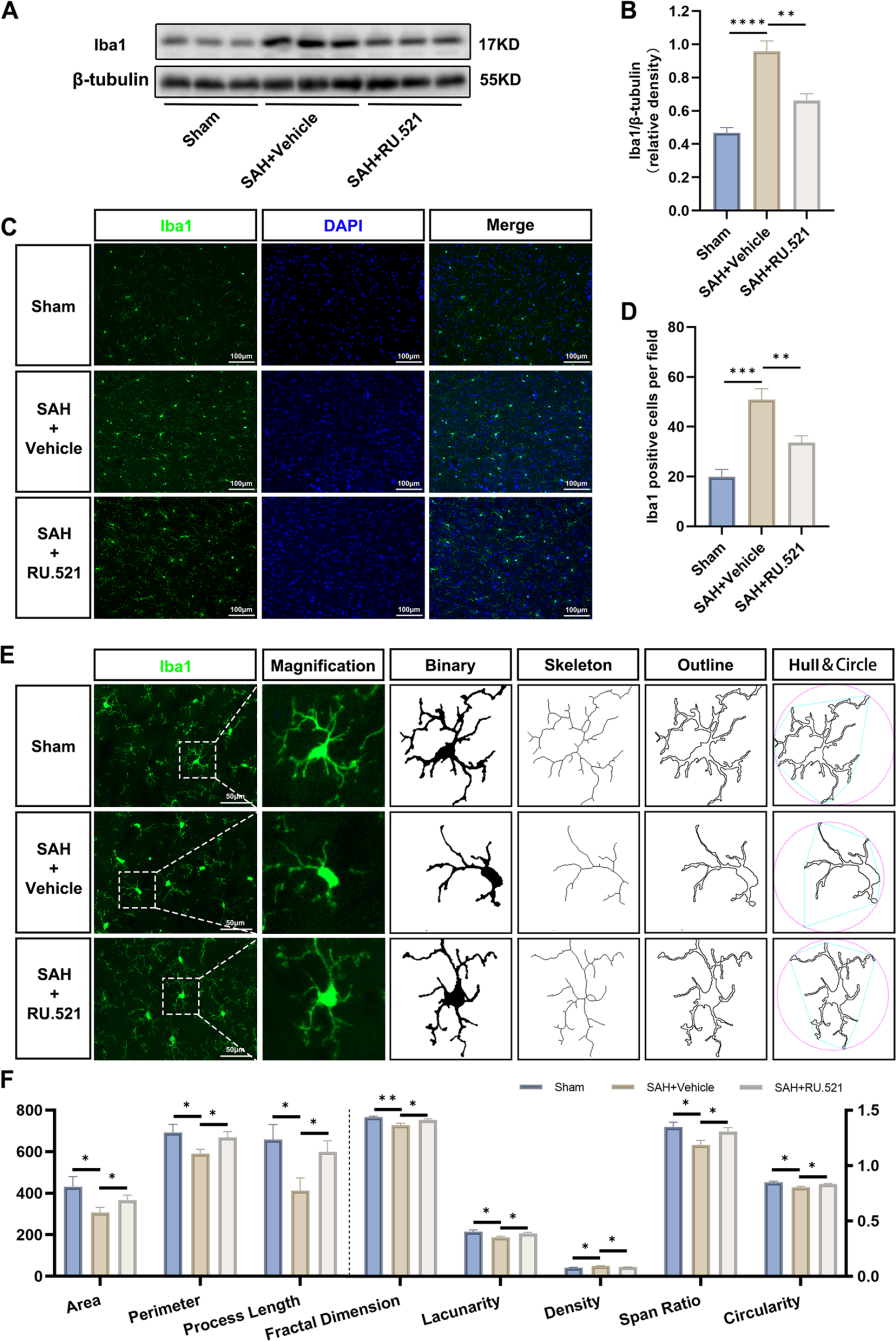
RU.521 blunted the over-activation of microglia after SAH. **A**, **B** Western blot bands and quantitative analysis for Iba1 expression. *n* = 6 per group. **C**, **D** Immunofluorescence staining and quantitative analysis of activated microglia (Iba1). Scale bar = 100 μm. *n* = 6 per group. **E** Representative images of activated microglia (green) for the measurement of morphological parameters and (**F**) quantitative analysis with the area, perimeter, process length, fractal dimension, lacunarity, density, span ratio, and circularity. Scale bar = 50 μm. *n* = 6 per group. *: *P* < 0.05, **: *P* < 0.01, ***: *P* < 0.001, and ****: *P* < 0.0001. ns, not significant

To investigate the response of microglia after SAH, we conducted western blot analysis for Iba1. The results of the analysis showed that the level of Iba1 in the SAH+Vehicle group was higher than that in the sham group. However, treatment with RU.521 resulted in a decrease in the elevated Iba1 level (Fig. 3A, B). The results of Iba1 immunofluorescence indicated that the number of activated microglia increased after SAH in comparison to the Sham group (Fig. 3C, D). RU.521 treatment significantly reduced the number of Iba1 positive microglia in the cerebral cortex (Fig. 3C, D). After SAH, the density of activated microglia increased morphologically. However, the area, perimeter, process length, fractal dimension, lacunarity, span ratio, and circularity were reduced compared to the Sham group (Fig. 3E, F). RU.521 treatment alleviated this alteration (*P* <0.05, Fig. 3E, F).

### RU.521 prompted microglial M1-to-M2 polarization and attenuated neuroinflammation via the cGAS/STING/NF-κB pathway

**Fig. 4:**
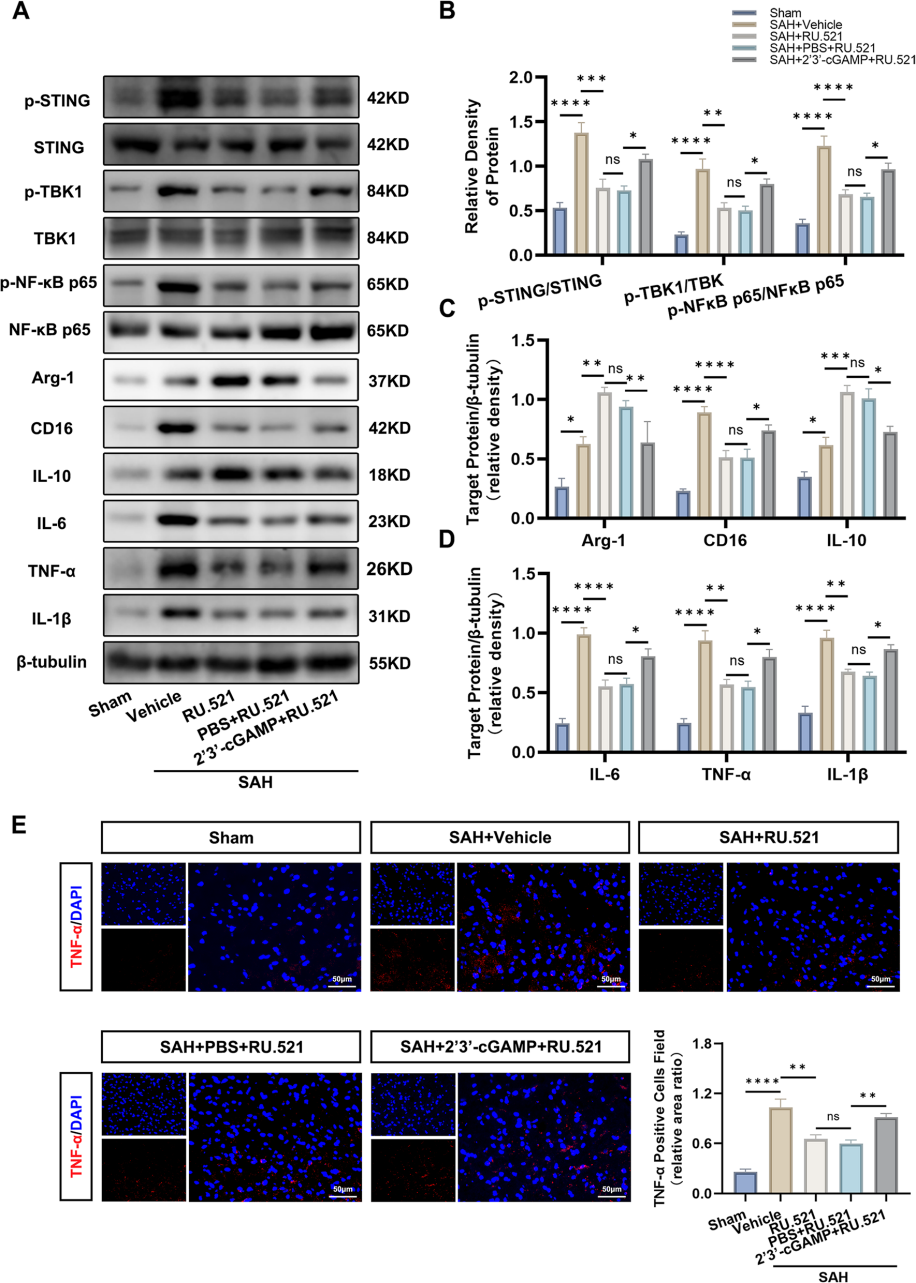
RU.521 prompted microglial M1-to-M2 polarization and attenuated neuroinflammation via the cGAS/STING/NF-κB pathway. **A**-**D** Western blot bands and quantitative analysis of the expression of p-STING, STING, p-TBK1, TBK1, p-NF-κB p65, NF-κB p65, Arg-1, CD16, IL-10, IL-6, TNF-α, and IL-1β. *n* = 6 per group. **E** The representative pictures of immunofluorescence and quantitative analysis for pro-inflammatory cytokine TNF-α. Scale bar = 50 μm. *n* = 6 per group. *: *P* < 0.05, **: *P* < 0.01, ***: *P* < 0.001, and ****: *P* < 0.0001. ns, not significant

Upon activation, microglia can adopt polarized phenotypes, which can either be classified as the proinflammatory M1 phenotype (marked by CD16, CD86, and iNOS) or the anti-inflammatory M2 phenotype (marked by CD206, CD163, and Arg-1) [15]. To delve deeper, 2’3’-cGAMP, which was a second messenger generated by activated cGAS, was utilized. In the western blot analysis, it was observed that there was an increase in p-STING/STING, p-TBK1/TBK1, and p-NF-κB p65/ NF-κB p65 in the SAH + Vehicle group when compared to the Sham group (Fig. 4A, B), the SAH + Vehicle group showed an increase in levels of Arg-1, CD16, IL-10, IL-6, TNF-α, and IL-1β (Fig. 4A, C, D). After RU.521 administration, the elevated level of p-STING/STING, p-TBK1/TBK1, p-NF-κB p65/ NF-κB p65, CD16, IL-6, TNF-α and IL-1β were partly reverse in SAH + RU.521 group. On the contrary, Arg-1 and IL-10 were still increased (Fig. 4A-D). Compared to the SAH + RU.521 group, the western blot analysis revealed that the SAH + 2’3’-cGAMP + RU.521 group exhibited partially restored levels of p-STING/STING, p-TBK1/TBK1, p-NF-κB p65/ NF-κB p65, CD16, IL-6, TNF-α, and IL-1β. Additionally, there were decreases in Arg-1 and IL-10 (Fig. 4A-D).

The results of the immunofluorescence test for the pro-inflammatory cytokine TNF-α indicated that the area of TNF-α-positive cells in the cortex after SAH was significantly increased compared to the sham group. The use of RU.521 reduced this alteration, however, the alteration was partly rescued in the SAH + 2’3’-cGAMP + RU.521 group (Fig. 4E).

### RU.521 improved the recovery of neurological function and dendritic spine densities

**Fig. 5:**
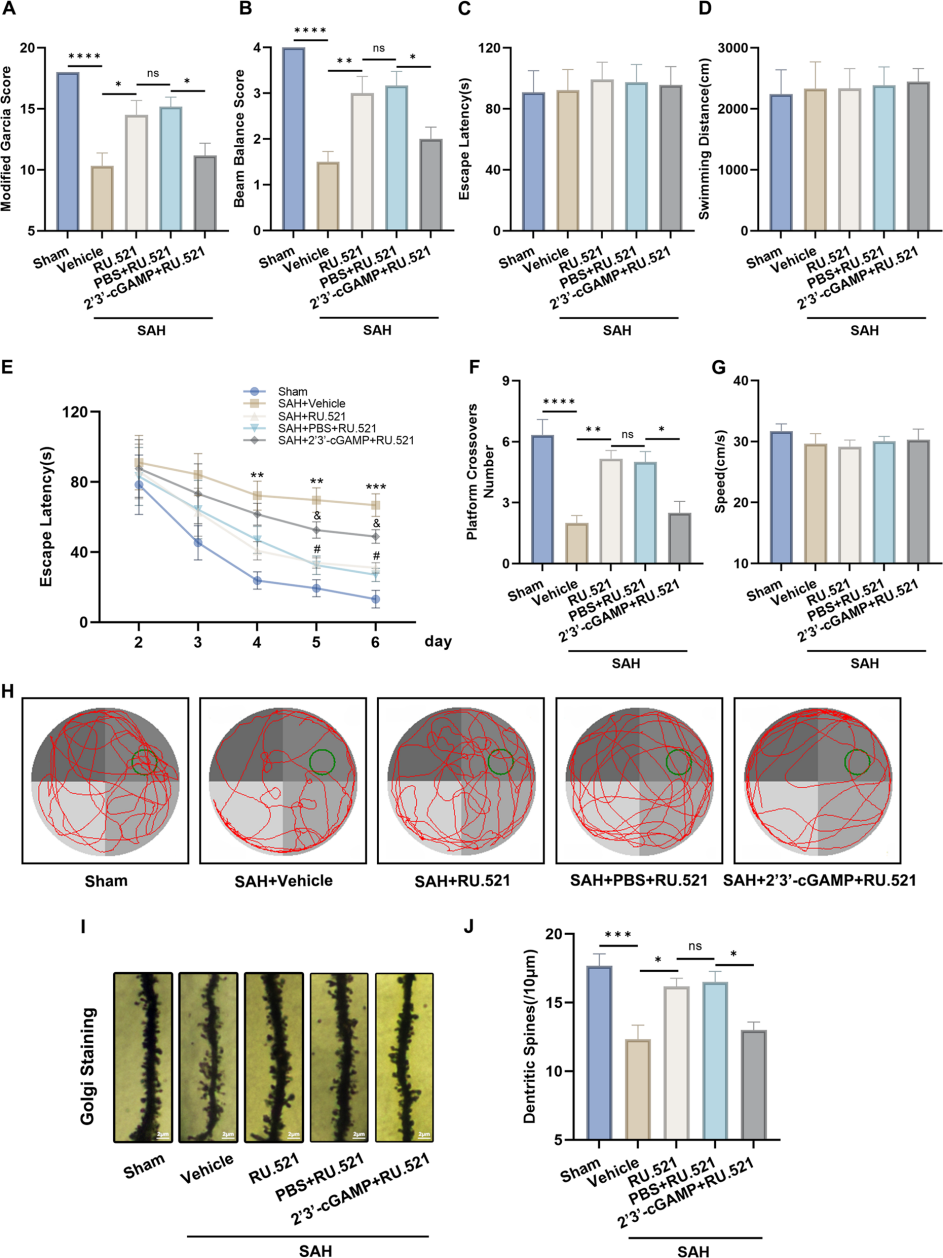
RU.521 promoted the recovery of neurological function and dendritic spine densities. **A**, **B** Analysis of the modified Garcia scores and beam balance scores of each group. *n* = 6 per group. **C**, **D** Escape latency and swim distance in the visible platform trial of the water maze test. *n* = 6 per group. **E** Escape latency in the hidden platform trial of the water maze test. *n* = 6 per group. **F**–**H** The times each animal crossed the original position of the platform, swimming speed, and swimming path in the probe trial of the water maze test. *n* = 6 per group. **I**, **J** Representative images of Golgi staining for dendritic spine densities and the analysis results. Scale bar = 2 μm. *n* = 6 per group. *: *P* < 0.05, **: *P* < 0.01, ***: *P* < 0.001, and ****: *P* < 0.0001. ^&^: *P* < 0.05, ^&&^: *P* < 0.01, ^&&&^: *P* < 0.001, and ^&&&&^: *P* < 0.0001. ^#^: *P* < 0.05, ^##^: *P* < 0.01, ^###^: *P* < 0.001, and ^####^: *P* < 0.0001. ns, not significant

Compared to the SAH + vehicle group, the SAH + RU.521 group showed significant improvement in modified Garcia scores and beam balance scores. However, the positive effects of RU.521 were partially present in the SAH + 2’3’-cGAMP + RU.521 group (Fig. 5A, B).

Spatial learning and memory were assessed using the Morris water maze test between days 22 to 28 following SAH. Results from the visible platform trial showed no significant difference in escape latency and swimming distance between the various groups (Fig. 5C, D). During the spatial learning trial, the SAH + vehicle group showed a significant increase in escape latency on days 4–6 compared to the Sham group. However, treatment with RU.521 partially restored this increase in the SAH + RU.521 group. The effect was nullified in the SAH + 2’3’-cGAMP + RU.521 group (Fig. 5E). During the probe trial, the swimming speed did not show significant difference among the groups. However, the SAH + vehicle group showed a significant reduction in the number of times each animal crossed the original position of the platform compared to the Sham group. The SAH + RU.521 group exhibited a performance improvement, whereas the SAH + 2’3’-cGAMP + RU.521 group showed a deficit in performance (Fig. 5F-H). Together, the results indicated that spatial learning and memory function was improved in the RU.521 treated group when compared to SAH-induced rats. However, the performance was significantly reduced in the SAH + 2’3’-cGAMP + RU.521 group.

The study evaluated dendritic spine densities in the second or third dendritic terminal branches of the hippocampus in different groups using Golgi staining. Results showed that the SAH+vehicle group had lower spine densities compared to the Sham group. However, the SAH+RU.521 group showed an improvement in spine density decline compared to the SAH+vehicle group. On the other hand, the rescue was impaired in the SAH+2’3’-cGAMP+RU.521 group (Figure 5I, J).

### RU.521 inhibited neuronal damage in vitro

**Fig. 6:**
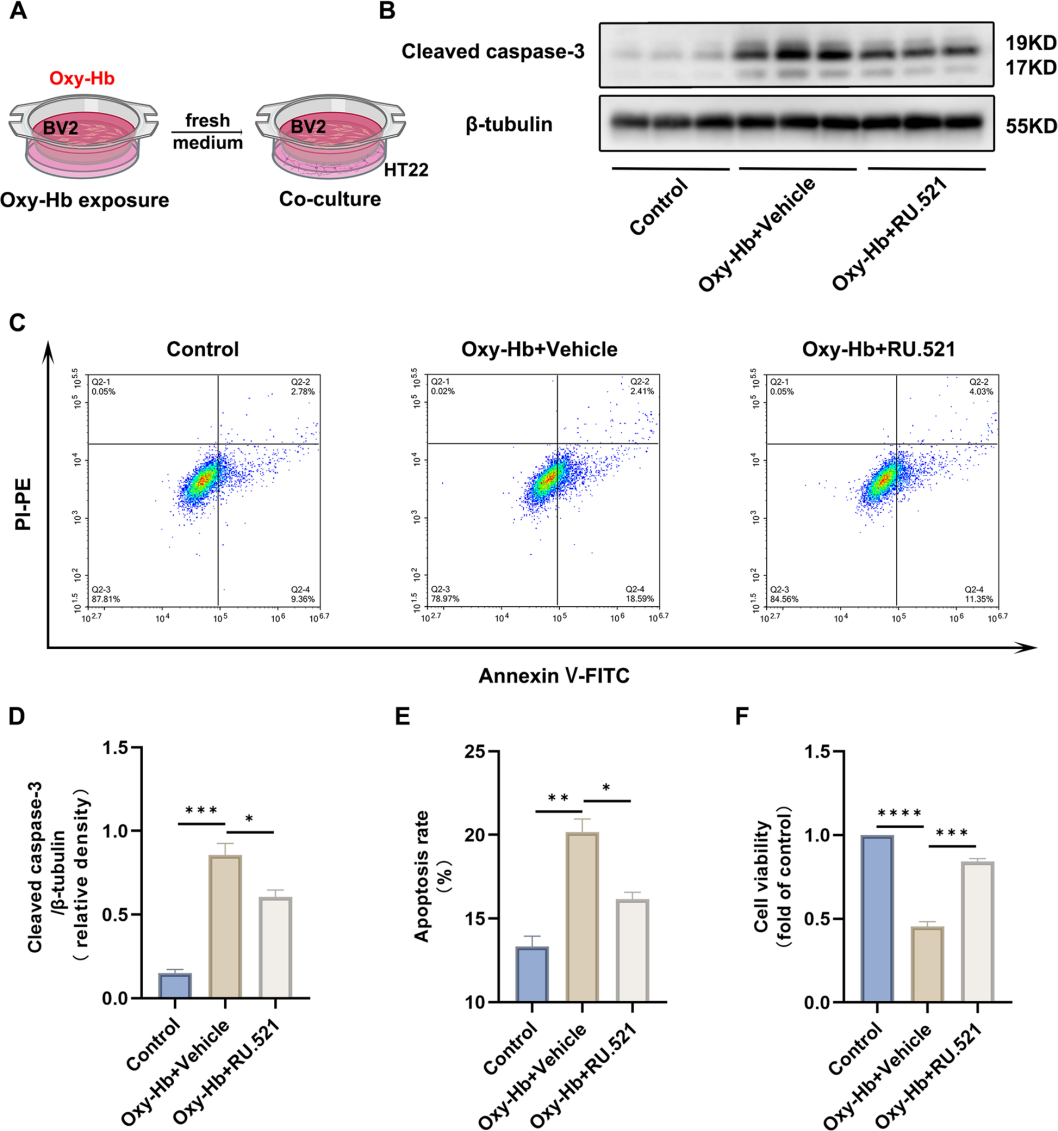
RU.521 inhibited neuronal damage in vitro. **A** Illustration of the co-culture system. **B**, **D** Representative western blot bands and quantitative analysis of the expression of cleaved-caspase 3 for TH22 cells. *n* = 3 per group. **C**, **E** Representative flow cytometry plots for HT22 cells. The apoptosis rate is calculated by the proportion of PI ^+^/Annexin V^+^ plus PI ^−^/Annexin V^+^. *n* = 3 per group. **F** Quantitative analysis of cell viability detected by CCK8 assays. *n* = 3 per group. *: *P* < 0.05, **: *P* < 0.01, ***: *P* < 0.001, and ****: *P* < 0.0001. ns, not significant

To better understand the mechanism in vitro, we introduced the microglia-neuron cocultures system for further research according to previous reports with some modifications [5, 36]. Pre-treated mouse BV2 microglial cells and mouse HT22 hippocampal neurons were co-cultured in Transwell plates (Corning, USA) to establish an in vitro model of SAH (Fig. 6A). The expression of apoptosis-related protein cleaved-caspase 3 was found to be increased in the Oxy-Hb + Vehicle group when compared to the control group in HT22 cells. However, the administration of RU.521 was able to reverse the changes induced by Oxy-Hb (Fig. 6B, D). In addition, flow cytometry analysis using Annexin V-FITC Apoptosis Detection Kit revealed an increase in the apoptosis rate of the Oxy-Hb + Vehicle group when compared to the control group. However, the administration of RU.521 partially reversed this change (Fig. 6C, E). In addition, the results of the CCK8 cell viability measurement also supported the above findings(Fig. 6F).

### RU.521 promoted microglial M1-to-M2 phenotypic polarization in vitro

**Fig. 7:**
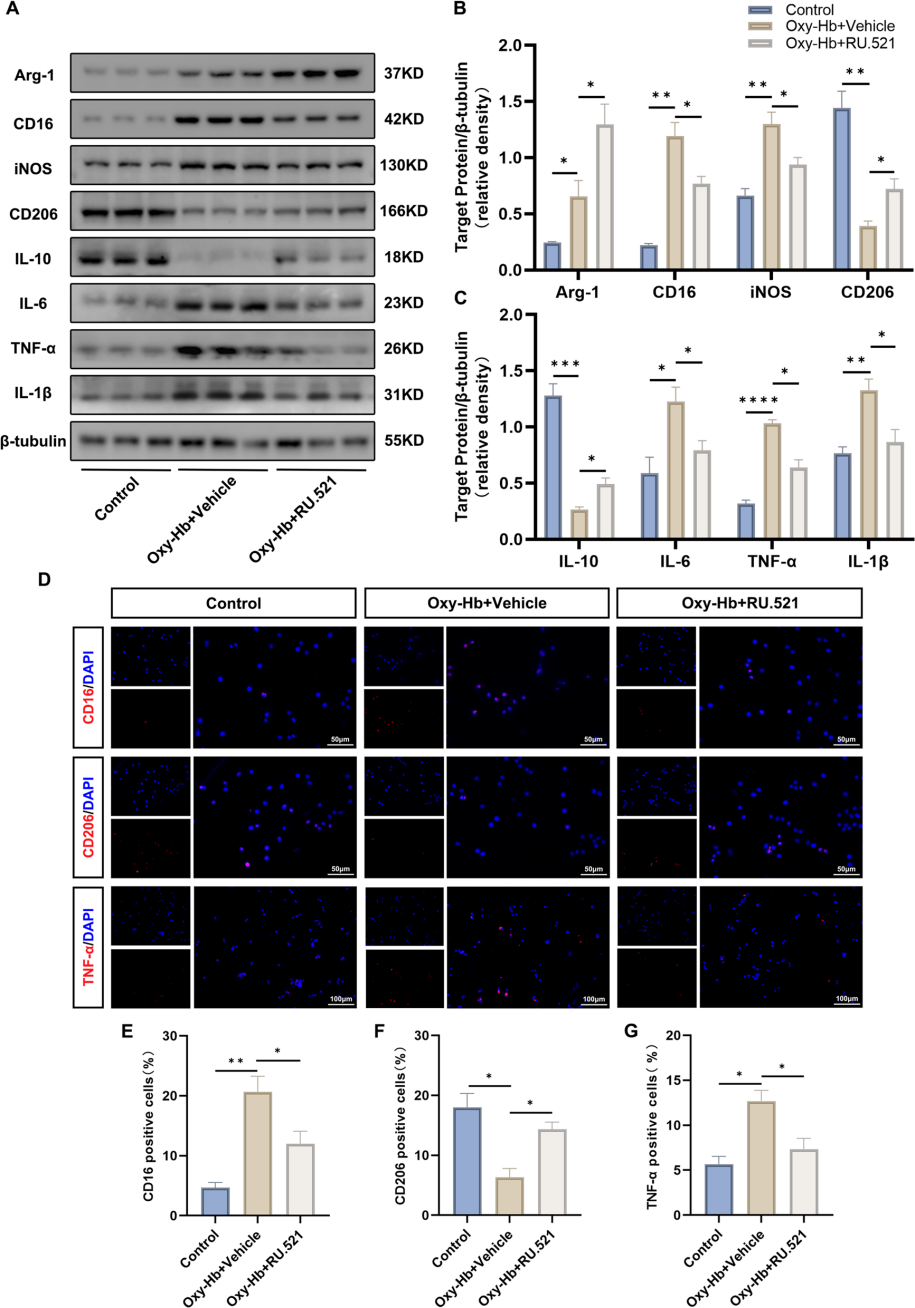
RU.521 promoted microglial M1-to-M2 phenotypic polarization in vitro. **A**-**C** Western blot bands and quantitative analysis for target protein expression in BV2 cells. *n* = 3 per group. **D**-**G** Representative images of immunofluorescence staining and quantitative analysis for CD16, CD206, and TNF-α. Scale bar = 50/100 μm. *n* = 3 per group. *: *P* < 0.05, **: *P* < 0.01, ***: *P* < 0.001, and ****: *P* < 0.0001. ns, not significant

BV2 cells showed an increase in protein expression of M2 phenotype-specific markers Arg-1 and M1 phenotype-specific markers CD16, and iNOS after exposure to Oxy-Hb compared to the control group. However, there was a decrease in M2 phenotype-specific markers CD206. Treatment with RU.521 increased the expression of Arg-1 and CD206, while the expression of CD16 and iNOS decreased (Fig. 7A, B).

In general, the M1 phenotypic microglia were pro-inflammatory, while the M2 phenotypic microglia were anti-inflammatory. Then we evaluated the level of cytokines among groups with western blot analysis. Compared to the control group, the Oxy-Hb group showed a significant decrease in the protein level of the anti-inflammatory cytokine IL-10, while the protein expression of the pro-inflammatory cytokines IL-6, TNF-α, and IL-1β increased. However, after RU.521 treatment, this trend improved (Fig. 7A, B).

Immunofluorescence staining confirmed the above results. The staining showed a significant increase in the proportion of CD16-positive cells and TNF-α-positive cells in the Oxy-Hb + Vehicle group compared to the control group. However, the proportion of CD206-positive cells showed a reverse trend. After treatment with RU.521 in the Oxy-Hb + RU.521 group, there was a significant decrease in the trend mentioned above, as shown in Fig. 7D-G. These findings demonstrated that RU.521 could induce microglial M1-to-M2 phenotypic polarization, which contributed to its anti-inflammatory effects.

### RU.521 inhibited microglial cGAS/STING pathway and the nuclear translocation of the p65 subunit of NF-κB in vitro

**Fig. 8:**
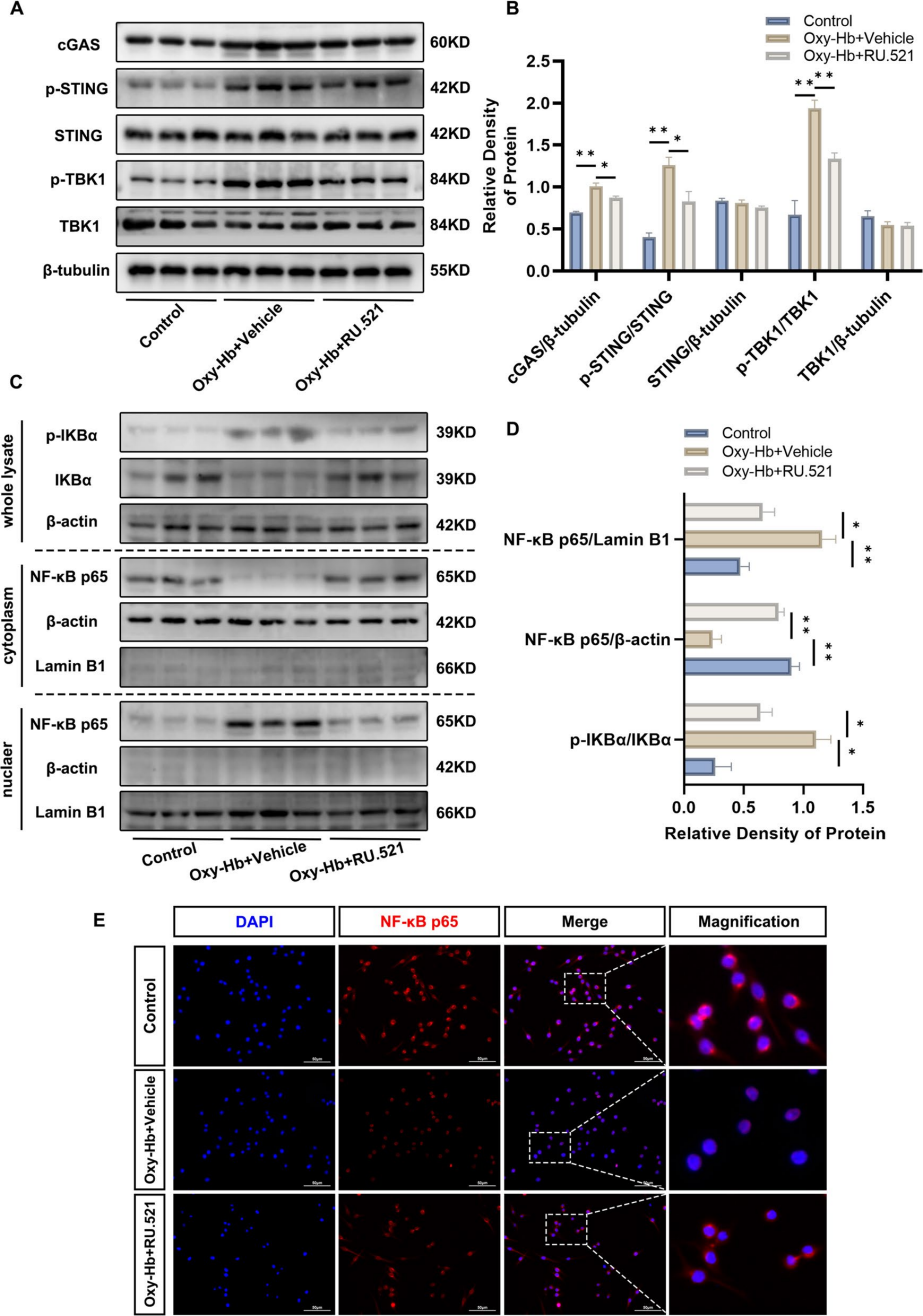
RU.521 inhibited microglial cGAS/STING pathway and the nuclear translocation of the p65 subunit of NF-κB in vitro*. ***A**, **B** Immunoblots analysis for the expression of cGAS, p-STING, STING, p-TBK1, and TBK1. *n* = 3 per group. **C**, **D** Immunoblots and quantitative analysis of the expression of p-IKBα and NF-kB p65. *n* = 3 per group. **E** Immunofluorescence staining for NF-kB p65 (red) and cell nuclei with DAPI (blue). Scale bar = 50 μm. *: *P* < 0.05, **: *P* < 0.01, ***: *P* < 0.001, and ****: *P* < 0.0001. ns, not significant

To further investigate the effect of RU.521 on microglial M1-to-M2 phenotypic polarization, we analyzed the dynamic changes of cGAS/STING pathway components in vitro using western blot analysis. The cGAS, p-STING/STING, and p-TBK1/TBK1 expression levels were significantly increased in the Oxy-Hb + Vehicle group compared to the control group. However, the increased levels were reduced after the administration of RU.521 (Fig. 8A, B).

NF-κB was a crucial nuclear transcription factor involved in neuroinflammation. To further investigate this, western bot analysis was utilized. The obtained results indicated that there were fewer cytoplasmic NF-κB p65 and more p-IKBα/IKBα and nuclear NF-κB p65 in the Oxy-Hb + Vehicle group compared to the control group. However, the administration of RU.521 suppressed the elevated p-IKBα/IKBα and nuclear translocation of the p65 subunit of NF-κB. (Fig. 8C, D). Furthermore, the nuclear translocation of the p65 subunit of NF-κB was confirmed by immunofluorescence staining in the Oxy-Hb + Vehicle group, and the above process was suppressed by RU.521 (Fig. 8E).

## Discussion

**Fig. 9:**
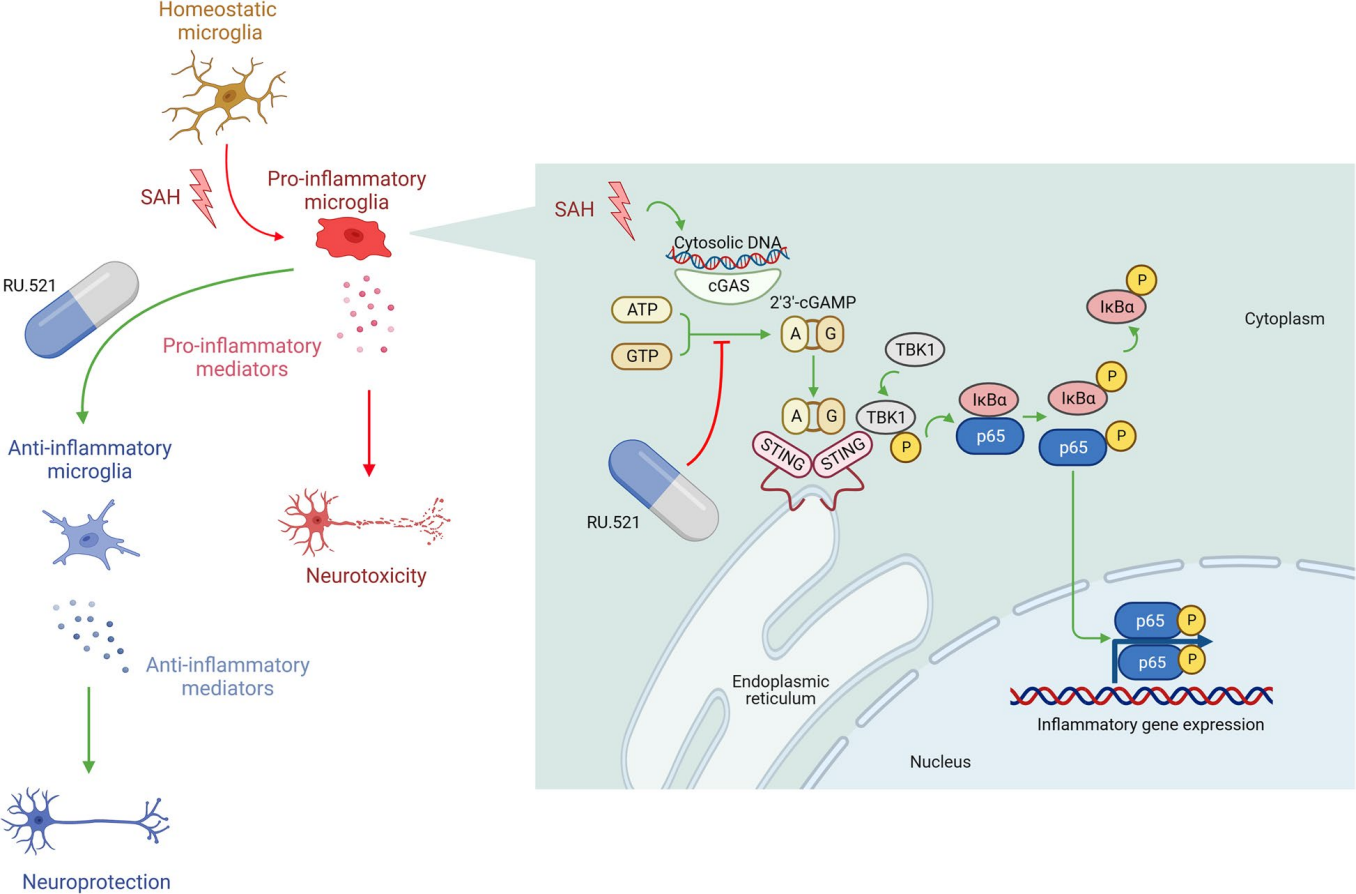
Schematic illustration of the possible mechanisms. Briefly, the activation of the cGAS/STING/NF-κB pathway was induced by subarachnoid hemorrhage, resulting in the expression of inflammatory genes and microglial activation. This activation led microglia to polarize towards the M1 phenotype, resulting in neuroinflammation through increased production of pro-inflammatory cytokines, which could ultimately worsen brain injury. RU.521 can regulate microglial polarization and reduce neuroinflammation, thereby mitigating these harmful effects. The figure was created with Biorender.com. Agreement number: TW25N2UE2X

In this study, we observed an increase in cGAS expression levels in rat brain tissue following subarachnoid hemorrhage (SAH). We found that inhibiting cGAS with the specific inhibitor RU.521 resulted in a reduction of brain water content and blood–brain barrier permeability. Furthermore, this treatment improved neurological deficits, impaired cognition, and dendritic spine densities after SAH. RU.521 was found to have a positive impact on neuronal apoptosis and microglial activation. Additionally, it was observed that RU.521 prompted microglial transformation from the M1 phenotype to the M2 phenotype, which led to a reduction in the production of TNF-α, IL-1β, and IL-6. Furthermore, RU.521 improved the decreased level of IL-10. The cGAS/STING/NF-κB pathway was found to mediate these effects, and it was noted that the therapeutic effects of RU.521 were partly abolished by 2’3’-cGAMP. In vitro, treatment with RU.521 significantly improved the cell viability of HT22 cells and reduced apoptosis. Additionally, it inhibited the nuclear translocation of the p65 subunit of NF-κB and prevented the transformation of BV2 cells into the M1 phenotype after exposure to Oxy-Hb. Furthermore, RU.521 promoted the switching of BV2 cells towards the M2 phenotype, which further confirmed the aforementioned findings after SAH in rats. Our study showed that neuroinflammation caused by the activation of microglia after SAH contributed to early brain injury. By inhibiting cGAS with RU.521, we were able to improve neurological outcomes and reduce neuroinflammation in early brain injury. This was achieved through the regulation of microglial polarization and neuroinflammation mediated by the cGAS/STING/NF-κB pathway (Fig. 9). We were the first to report that the cGAS selective inhibitor RU.521 ameliorates subarachnoid hemorrhage-induced brain injury via regulating microglial polarization and neuroinflammation mediated by the cGAS/STING/NF-κB pathway.

According to reports, cGAS was a cytoplasmic nucleic acid sensor that can recognize double-stranded DNA (dsDNA) with the widest range of specificity. It can bind to dsDNA in a manner that was not dependent on sequence specificity [37]. The main function of cGAS was to activate the downstream adaptor protein STING, which in turn induced the production and release of IFN. This mechanism, known as cGAS-STING signaling, was highly conserved throughout evolution and played a crucial role in immunity [38, 39]. In addition to responding to foreign DNA, the system also responded to self-DNA, which was closely linked to neuroinflammation in cases of ischemic stroke [40, 41], amyotrophic lateral sclerosis [42], neonatal hypoxic-ischemic encephalopathy [27], and traumatic brain injury [23, 43]. RU.521 was a potent and selective cGAS inhibitor that effectively suppresses cGAS enzyme activity and downstream inflammatory events in both mice and humans [24, 44]. Our study demonstrated that the cGAS-STING pathway was activated after SAH, which resulted in the nuclear translocation of the p65 subunit of NF-κB and the production of proinflammatory mediators, leading to microglial activation. We found that inhibiting cGAS with RU.521 offered neuroprotection by regulating microglial polarization and reducing neuroinflammation.

Microglia, which were resident immune cells in the central nervous system (CNS), were responsible for immune defense and modulation of inflammation [12, 15]. Despite the occurrence of blood–brain barrier disruption following SAH, recent studies had shown that the initial neuroinflammatory response was primarily triggered by resident microglia, as opposed to infiltrating macrophages [45]. Microglia responded promptly to SAH, while peripheral infiltrating macrophages were not detected in the brain tissue until 72 h later [46, 47]. Zheng's study demonstrated that while the blood–brain barrier disruption after SAH permitted peripheral macrophages to enter the CNS, Iba1-positive cells were primarily derived from resident microglia [18]. Once activated, resident microglia rapidly proliferated and accumulated at the pathology site, which performed phagocytosis of cells and debris, production of cytokines and chemokine leading to neuroinflammation [48]. Neuroinflammation, especially microglia-mediated, contributed to injury expansion and brain damage in SAH [18, 49]. Morphological changes in the microglia were known to reflect their activation state [25, 30]. Homeostatic microglia had a highly ramified morphology. When activated, microglia can change such as developing enlarged cell bodies, shortened processes, and polarization into two phenotypes: the proinflammatory M1 phenotype (marked by CD16, CD86, and iNOS) or the anti-inflammatory M2 phenotype (marked by CD206, CD163, and Arg-1) [16, 30]. Numerous studies had provided evidence that an elevation in M2 phenotype microglia following SAH was linked to a more favorable neurological outcome, which indicated that a promising approach for microglia-mediated neuroinflammation in SAH was regulating microglial phenotype from M1 to M2 [18, 50, 51]. However, the specific mechanisms and modulators were not completely understood [52]. To further evaluate the microglial response status following SAH, we utilized skeleton analysis and fractal analysis with immunofluorescence for Iba1 to assess microglial morphology. The study found that RU.521 treatment resulted in significant relief in microglial morphology, as evidenced by changes in morphological parameters such as density, process length, fractal dimension, lacunarity, area, perimeter, span ratio, and circularity. Additionally, RU.521 increased the expression of M2 phenotype markers Arg-1 and CD206, while decreasing the expression of M1 phenotype markers iNOS and CD16. Furthermore, the administration of RU.521 can reduce the levels of pro-inflammatory mediators such as TNF-α, IL-1β, and IL-6, while increasing the levels of anti-inflammatory mediator IL-10. Additionally, RU.521 can effectively reduce neuronal apoptosis following SAH, potentially due to its ability to suppress the production of inflammatory cytokines from activated microglia. Long before DNA was recognized as genetic material, it was known to be responsible for immune responses [53]. Combined with cGAS as a cytoplasmic nucleic acid sensor, our results not only linked activation of the cGAS-STING pathway to polarized microglia-mediated neuroinflammation in SAH for the first time but also supported the hypothesis that mislocalized DNA, such as cytoplasmic dsDNA and mitochondrial DNA, contributed to microglial polarization and neuroinflammation. Although this hypothesis needed to be confirmed with further research, it advanced the current understanding of the mechanisms of microglia-mediated neuroinflammation in SAH.

NF-kB was an important nuclear transcription factor related to the inflammatory response [54–56]. Upon activation, the inhibition protein IkB was phosphorylated and the free NF-kB complex translocated into the nucleus where it bound to DNA, leading to the transcription of inflammatory cytokines [55]. The findings of our study indicated that RU.521 can impede the phosphorylation of IkBα and the nuclear translocation of the p65 subunit of NF-κB.

The current study provided initial insight into the potential of RU.521 in reducing brain injury caused by SAH. This effect was achieved through the regulation of microglial polarization and neuroinflammation, which was mediated by the cGAS/STING/NF-κB pathway. However, it was important to acknowledge the limitations of our research. Firstly, the regulatory potential of the above pathway on other cell types, such as astrocytes, had not been evaluated and warranted further research. Secondly, it was important to note that in vitro stimulation with oxyhemoglobin did not entirely replicate the SAH process in vivo. Lastly, it was worth noting that endogenic dsDNA included both mitochondrial DNA (mtDNA) and disintegrated nuclear DNA. It was unclear which cell type was the source for endogenic dsDNA and whether cGAS sensed one or both of the above components in SAH.

## Conclusions

The study demonstrated that early brain injury resulting from subarachnoid hemorrhage was linked to neuroinflammation caused by microglia activation. The use of RU.521 to inhibit cGAS can improve neurological outcomes and reduce neuroinflammation by regulating microglial polarization and the cGAS/STING/NF-κB pathway. RU.521 could be a promising candidate for treating neuroinflammatory pathology following SAH.

### Supplementary Information

**Additional file 1: Supplementary Figure S1.** Experimental design and groups. SAH, subarachnoid hemorrhage; WB, western blot; IF, immunofluorescence; RU.521, selective small molecule inhibitor for cGAS; Vehicle, 1% DMSO + corn oil; TUNEL: terminal deoxynucleotidyl transferase-mediated dUTP nick end labeling; MWM, Morris water maze; 2’3’-cGAMP, a second messenger converted by activated cGAS; PBS, phosphate-buffered saline; Oxy-Hb, oxyhemoglobin; Annexin V-FITC, Annexin V-FITC Apoptosis Detection Kit; CCK-8, Cell Counting Kit-8. The figure was created with Biorender.com. Agreement number: HV25N2UEYC. **Supplementary Figure S2.** Representative images for brain tissues and SAH grading scores for each group. (A) This figure displayed representative brain images for sham (left) and SAH (right) conditions in rats, where the SAH image showed blood clots in the subarachnoid space. Additionally, an illustration of six parts on the ventral surface of the brain after SAH in rats was presented on the right. (B) The SAH grading scores for each group 24 h after SAH was shown in panel B. *: *P* < 0.05 vs. Sham. Vehicle, 1% DMSO + corn oil; PBS, phosphate-buffered saline. **Supplementary Table S1.** The grading system for SAH. **Supplementary Table S2.** Modified Garcia score. **Supplementary Table S3.** Beam balance test. **Supplementary Table S4.** Antibodies used in this study. **Supplementary Table S5.** Distribution of animals according to different groups and mortality rate.

**Additional file 2. **Uncropped Western bot gel images.

## Data Availability

The data supporting the findings of this study will be available from the corresponding author upon reasonable request.

## Abbreviations

Arg-1: Arginase-1
BBB: Blood-brain barrier
cGAS: Cyclic GMP–AMP synthase
DMSO: Dimethyl sulfoxide
EBI: Early brain injury
Iba1: Ionized calcium-binding adaptor molecule 1
IL-10: Interleukin 10
IL-1β: Interleukin-1β
IL-6: Interleukin 6
iNOS: Inducible nitric oxide synthase
NeuN: Neuronal nuclei
NF-κB: Nuclear factor kappa-B
Oxy-Hb: Oxyhemoglobin
PBS: Phosphate buffer solution
SAH: Subarachnoid hemorrhage
STING: Stimulator of interferon genes
TBK1: TANK binding kinase 1
TNF-α: Tumor necrosis factor-alpha
