# Correction: RU.521 mitigates subarachnoid hemorrhage-induced brain injury via regulating microglial polarization and neuroinflammation mediated by the cGAS/STING/NF-κB pathway

**DOI:** 10.1186/s12964-024-01772-x

**Published:** 2024-08-06

**Authors:** Jiang Shao, Yuxiao Meng, Kaikun Yuan, Qiaowei Wu, Shiyi Zhu, Yuchen Li, Pei Wu, Jiaolin Zheng, Huaizhang Shi

**Affiliations:** 1 Department of Neurosurgery, the First Afliated Hospital of Harbin Medical University, Youzheng Street 23#, Nangang District, Harbin, Heilongjiang Province 150001 China; 2 Department of Neurology, Second Afliated Hospital of Harbin Medical University, Xuefu Road 246#, Nangang District, Harbin, Heilongjiang Province 150001 China

**Correction: Cell Commun Signal 21, 264 (2023)**

10.1186/s12964-023-01274-2

Following publication of the original article [1], the authors reported an error in Fig. 1A, whereby the western blot band of β-tubulin in Fig. 1A was misused unintentionally, which overlapped with the band in Fig. 4A. This error occurred during the figure preparation.

The incorrect Fig. 1 is

**Fig. 1:**
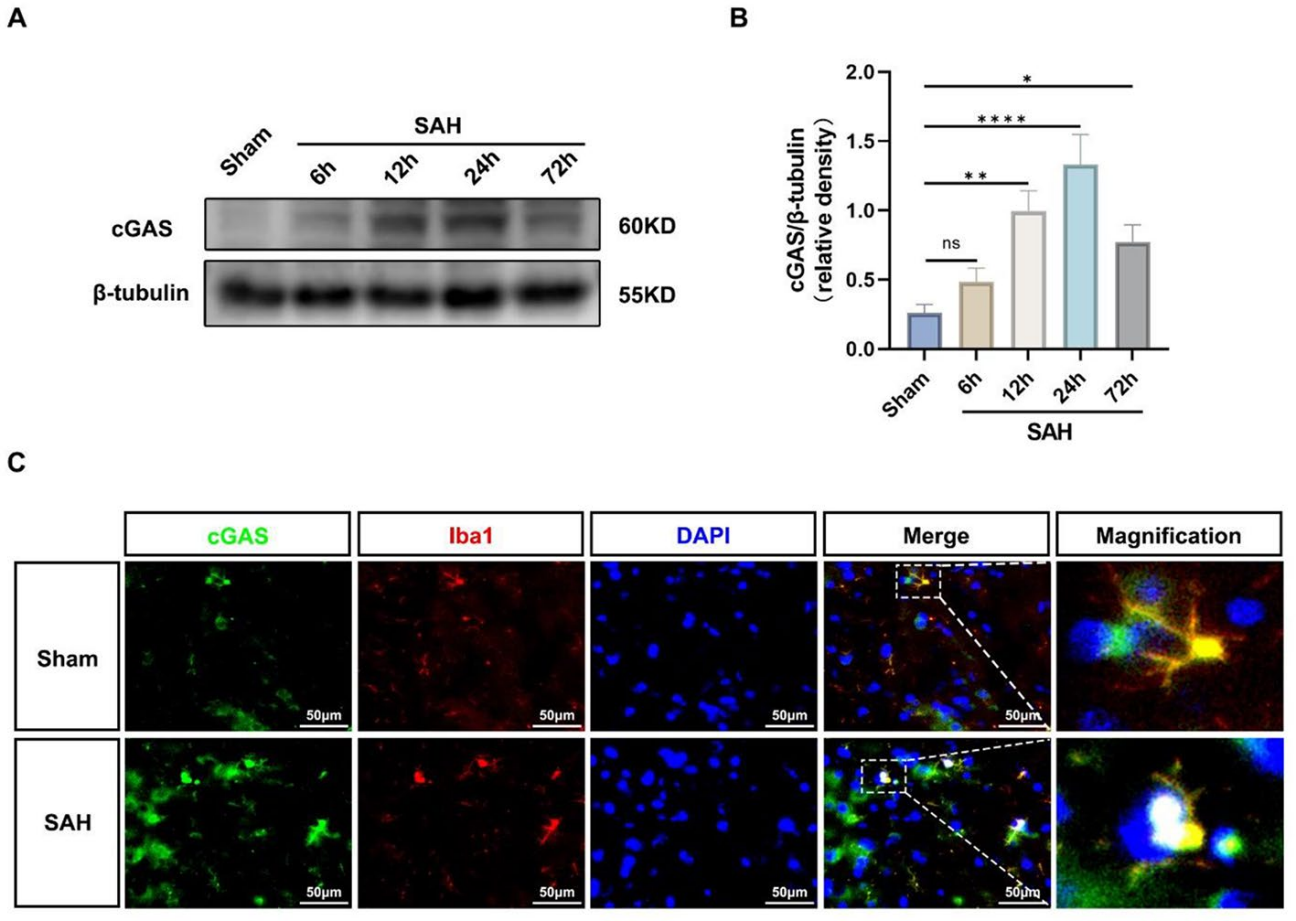
Expression changes of cGAS and microglial localization. **A**, **B** Representative Western blot bands of time course and quantitative analysis for cGAS. *n* = 6 per group. *: *P* < 0.05, **: *P* < 0.01, ***: *P* < 0.001, and ****: *P* < 0.0001. ns, not significant. **C** Representative images of double immunofluorescence staining for cGAS (green) with microglia (Iba1, red) at 24 h after SAH. Cell nuclei were counterstained with DAPI (blue). Scale bar = 50 μm. *n* = 6 per group

The correct Fig. 1 is:

**Fig. 1:**
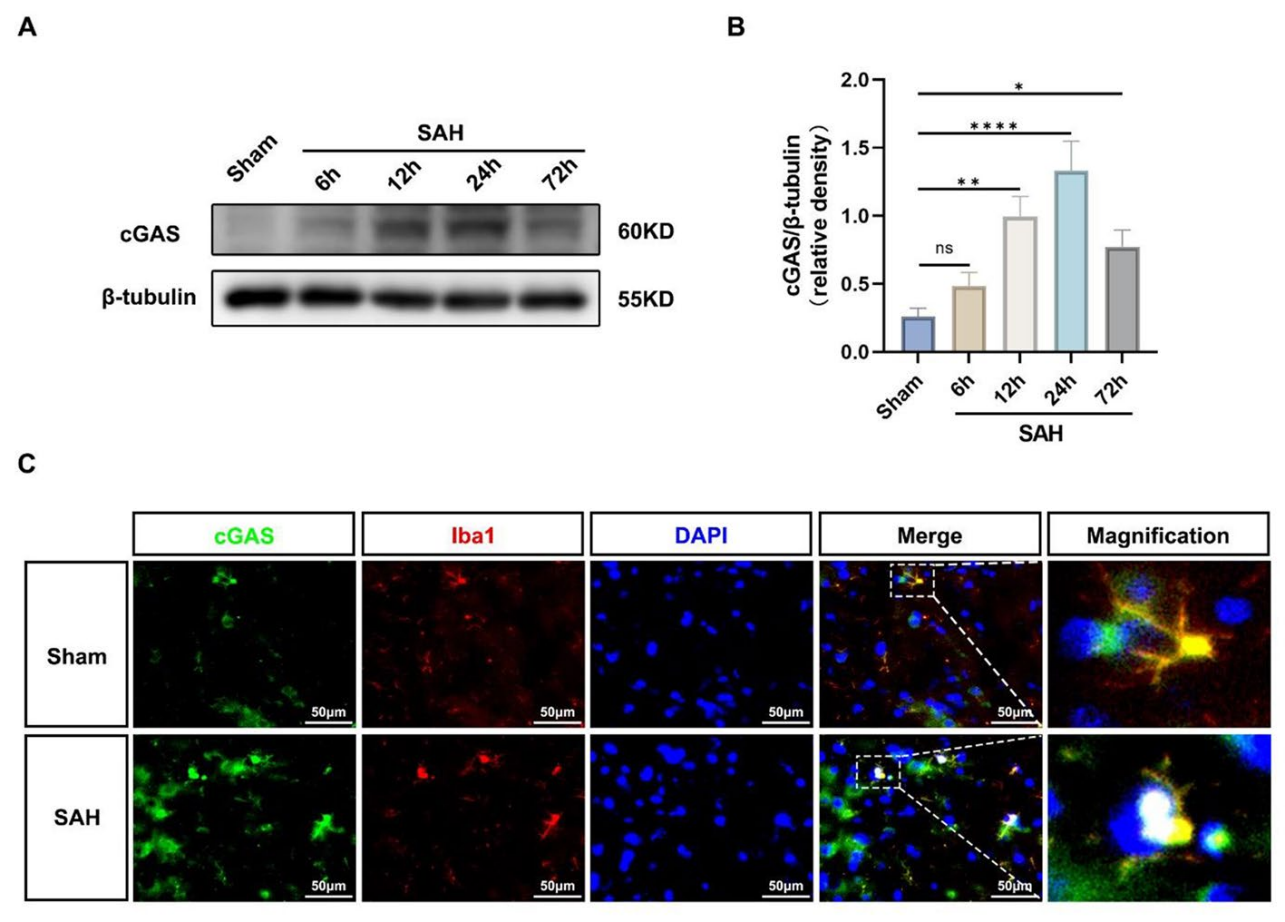
Expression changes of cGAS and microglial localization. **A**, **B** Representative Western blot bands of time course and quantitative analysis for cGAS. *n* = 6 per group. *: *P* < 0.05, **: *P* < 0.01, ***: *P* < 0.001, and ****: *P* < 0.0001. ns, not significant. **C** Representative images of double immunofluorescence staining for cGAS (green) with microglia (Iba1, red) at 24 h after SAH. Cell nuclei were counterstained with DAPI (blue). Scale bar = 50 μm. *n* = 6 per group

Further to this, please note Fig. 1A for β-tubulin related to Fig. 1A in the original supplementary file 2 was also incorrect. The complete updated supplementary file is provided as “Supplementary Material 2” in this correction article.

### Electronic supplementary material

Below is the link to the electronic supplementary material.

Supplementary Material 2: Uncropped Western bot gel images.
