# Supplementary material for: RU.521 mitigates subarachnoid hemorrhage-induced brain injury via regulating microglial polarization and neuroinflammation mediated by the cGAS/STING/NF-κB pathway
Source: Cell Commun Signal. 2023 Sep 28;21:264. doi: 10.1186/s12964-023-01274-2 (PMC10537158; doi:10.1186/s12964-023-01274-2)
Supplement: Supplementary file 2 — Additional file 1: Supplementary Figure S1. Experimental design and groups. SAH, subarachnoid hemorrhage; WB, western blot; IF, immunofluorescence; RU.521, selective small molecule inhibitor for cGAS; Vehicle, 1% DMSO + corn oil; TUNEL: terminal deoxynucleotidyl transferase-mediated dUTP nick end labeling; MWM, Morris water maze; 2’3’-cGAMP, a second messenger converted by activated cGAS; PBS, phosphate-buffered saline; Oxy-Hb, oxyhemoglobin; Annexin V-FITC, Annexin V-FITC Apoptosis Detection Kit; CCK-8, Cell Counting Kit-8. The figure was created with Biorender.com. Agreement number: HV25N2UEYC. Supplementary Figure S2. Representative images for brain tissues and SAH grading scores for each group. (A) This figure displayed representative brain images for sham (left) and SAH (right) conditions in rats, where the SAH image showed blood clots in the subarachnoid space. Additionally, an illustration of six parts on the ventral surface of the brain after SAH in rats was presented on the right. (B) The SAH grading scores for each group 24 h after SAH was shown in panel B. *: P < 0.05 vs. Sham. Vehicle, 1% DMSO + corn oil; PBS, phosphate-buffered saline. Supplementary Table S1. The grading system for SAH. Supplementary Table S2. Modified Garcia score. Supplementary Table S3. Beam balance test. Supplementary Table S4. Antibodies used in this study. Supplementary Table S5. Distribution of animals according to different groups and mortality rate. [file 12964_2023_1274_MOESM1_ESM.docx]

**Additional file 1 for**

**RU.521 mitigates subarachnoid hemorrhage-induced brain injury via regulating microglial polarization and neuroinflammation mediated by the cGAS/STING/NF-κB pathway**

Jiang Shao^1^, Yuxiao Meng^1^, Kaikun Yuan^1^, Qiaowei Wu^1^, Shiyi Zhu^1^, Yuchen Li^1^, Pei Wu^1^, Jiaolin Zheng^2*^, and Huaizhang Shi^1*^

^1^ Department of Neurosurgery, the First Affiliated Hospital of Harbin Medical University, Youzheng Street 23#, Nangang District, Harbin, 150001, Heilongjiang Province, China

^2^ Department of Neurology, the Second Affiliated Hospital of Harbin Medical University, Xuefu Road 246#, Nangang District, Harbin, 150001, Heilongjiang Province, China

**This file includes:**

**Supplementary Figure S1 to S2**

Supplementary Figure S1. Experimental design and groups.

Supplementary Figure S2. Representative images for brain tissues and SAH grading scores for each group.

**Supplementary Table S1 to S5**

Supplementary Table S1. The grading system for SAH.

Supplementary Table S2. Modified Garcia score.

Supplementary Table S3. Beam balance test.

Supplementary Table S4. Antibodies used in this study.

Supplementary Table S5. Distribution of animals according to different groups and mortality rate.

**Supplementary Figures:**

**
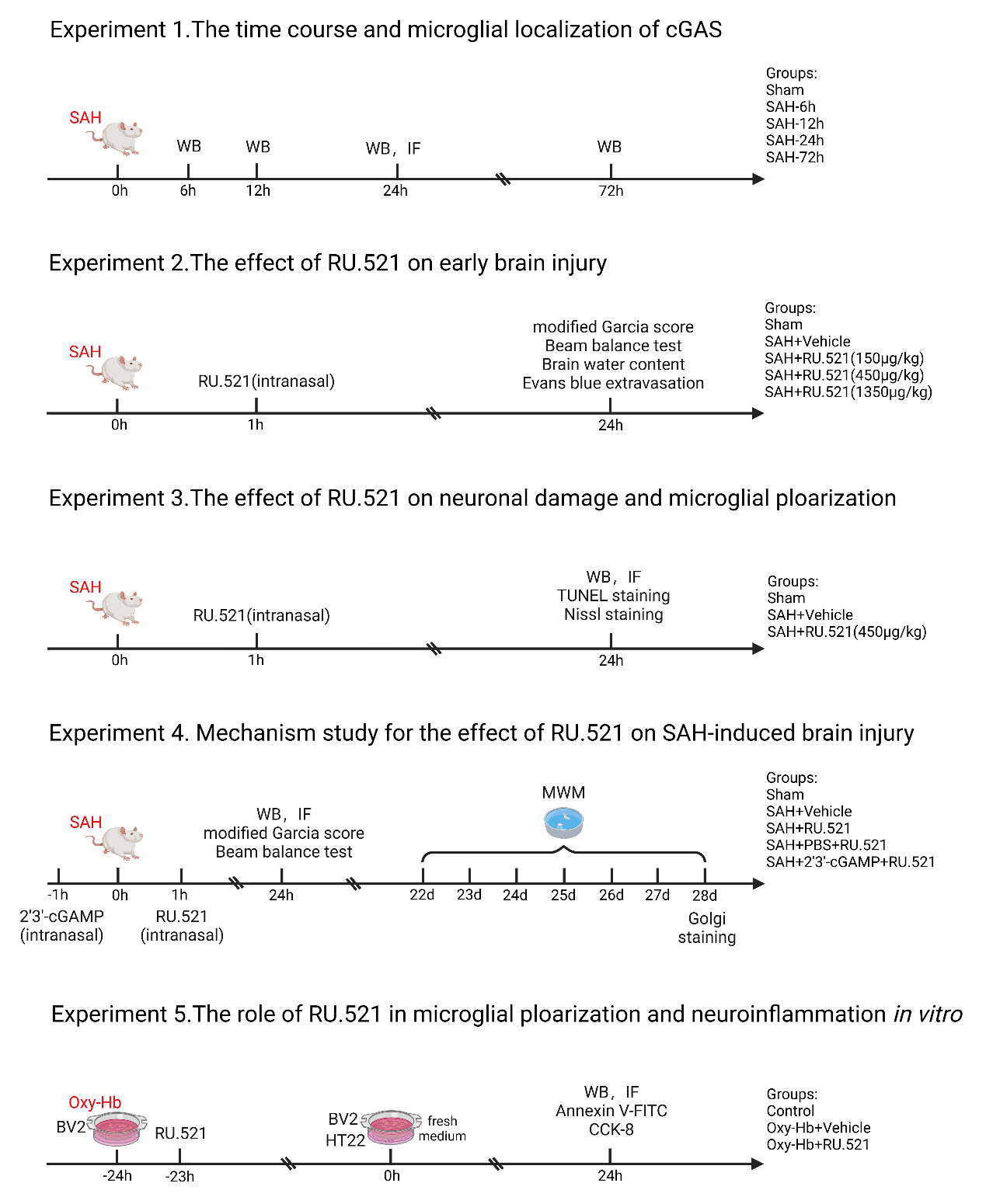
**

**Supplementary Figure S1:** **Experimental design and groups**. SAH, subarachnoid hemorrhage; WB, western blot; IF, immunofluorescence; RU.521, selective small molecule inhibitor for cGAS; Vehicle, 1% DMSO + corn oil; TUNEL: terminal deoxynucleotidyl transferase-mediated dUTP nick end labeling; MWM, Morris water maze; 2’3’-cGAMP, a second messenger converted by activated cGAS; PBS, phosphate-buffered saline; Oxy-Hb, oxyhemoglobin; Annexin V-FITC, Annexin V-FITC Apoptosis Detection Kit; CCK-8, Cell Counting Kit-8. The figure was created with Biorender.com. Agreement number: HV25N2UEYC.


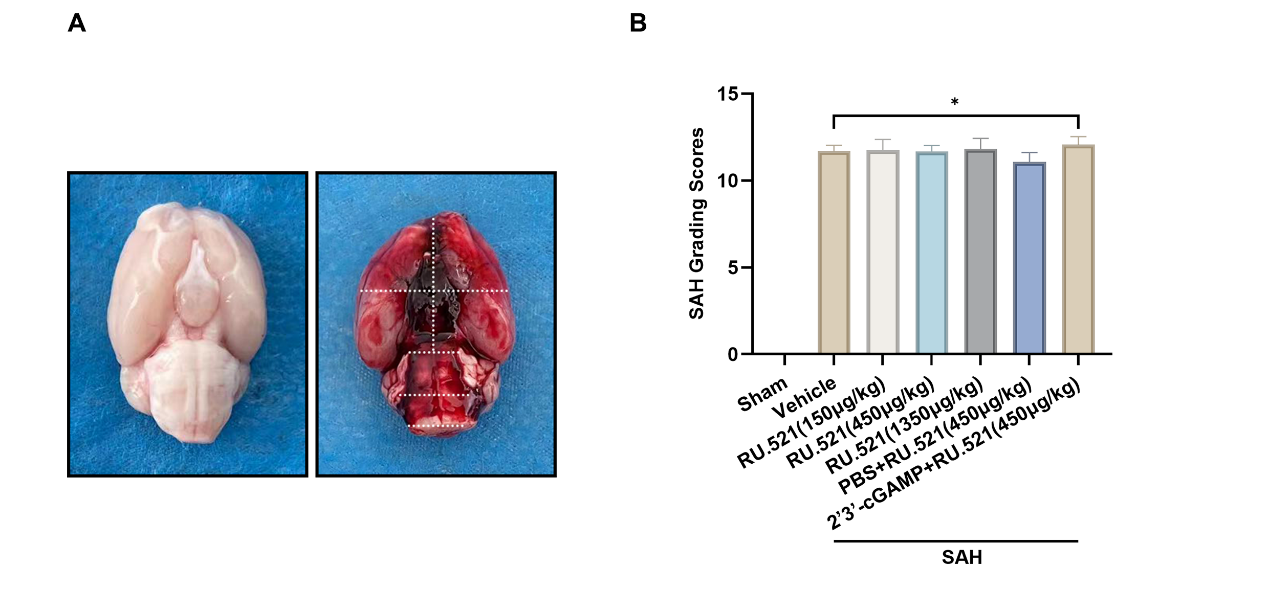


**Supplementary Figure S2: Representative images for brain tissues and SAH grading scores for each group.** (A) This figure displayed representative brain images for sham (left) and SAH (right) conditions in rats, where the SAH image showed blood clots in the subarachnoid space. Additionally, an illustration of six parts on the ventral surface of the brain after SAH in rats was presented on the right. (B) The SAH grading scores for each group 24 h after SAH was shown in panel B. *: *P* < 0.05 *vs.* Sham. Vehicle, 1% DMSO + corn oil; PBS, phosphate-buffered saline.

**Supplementary Tables：**

**Supplementary Table S1: The grading system for SAH.**

| **Score** | **Description** |
| --- | --- |
| 0 | no subarachnoid blood |
| 1 | minimal subarachnoid blood |
| 2 | moderate blood clot with recognizable arteries |
| 3 | blood clot obliterating all arteries within the part |

**Supplementary Table S2: Modified Garcia score.**

| **Test** | **Score** | | | |
| --- | --- | --- | --- | --- |
|  | **0** | **1** | **2** | **3** |
| Spontaneous Activity (in a cage for 5 min) | No movement | Barely moves position | Moves but does not approach at least three sides of the cage | Moves and approaches at least three sides of the cage |
| Spontaneous movements of all limbs | No movement | Slight movement of limbs | Moves all limbs but slowly | Move all limbs same as pre-SAH |
| Movements of forelimbs (outstretching while held by the tail) | No movement | Slight outreaching | Outreach is limited and less than pre-SAH | Outreach same as pre-SAH |
| Climbing the wall of the wire cage | - | Fails to climb | Climbs weakly | Normal climbing |
| Reaction to touch on both sides of the trunk | - | No response | Weak response | Normal response |
| Response to vibrissae touch | - | No response | Weak response | Normal response |

**Supplementary Table S3: Beam balance test.**

| **Score** | **Description: Beam walking (60 sec)** |
| --- | --- |
| 0 | No walking and falls off |
| 1 | No walking but remains on beam |
| 2 | Walking but falls off |
| 3 | Walking less than 20 cm |
| 4 | Walking beyond 20 cm |

**Supplementary Table S4: Antibodies used in this study.**

| **Antibody** | **Manufacturer** | **Catalog number** | **Dilution** |
| --- | --- | --- | --- |
| anti-cGAS | Santa Cruz | sc-515777 | 1: 500 |
| anti-STING | Proteintech | 19851-1-AP | 1:1000 |
| anti-p-STING | Abmart | TA7416s | 1:500 |
| anti-TBK1 | Abmart | TD7026s | 1:1000 |
| anti-p-TBK1 | Abmart | TA8190s | 1:1000 |
| anti-NF-kB-p65 | Cell Signaling Tech | #8242 | 1:1000 |
| anti-p-NF-kB p65 | Cell Signaling Tech | #3033 | 1:1000 |
| anti-IKBα | Abcam | ab32518 | 1:1000 |
| anti-p-IKB-α | Abcam | ab133462 | 1:5000 |
| anti-Iba1 | Abcam | ab178846 | 1:2000 |
| anti-Arg-1 | Abcam | ab96183 | 1:1000 |
| anti-iNOS | Abcam | ab178945 | 1:1000 |
| anti-CD206 | Abmart | TD4149 | 1:1000 |
| anti-CD16 | Abmart | T59545 | 1:1000 |
| anti-IL-6 | ABclonal | A11115 | 1:1000 |
| anti-IL-1β | ABclonal | A20529 | 1:1000 |
| anti-IL-10 | ABclonal | A2171 | 1:1000 |
| anti-TNF-α | ABclonal | A11534 | 1:1000 |
| anti-cleaved caspase-3 | Abcam | ab214430 | 1:500 |
| anti-Lamin B1 | Proteintech | 12987-1-AP | 1:1000 |
| anti-β-tubulin | Proteintech | 10068-1-AP | 1:1000 |
| anti-β-actin | Abcam | ab8226 | 1:2000 |
| Goat anti-Mouse IgG (H+L) Secondary Antibody, HRP | Invitrogen | 31430 | 1:5000 |
| Goat anti-Rabbit IgG (H+L) Secondary Antibody, HRP | Invitrogen | 31460 | 1:5000 |
| Anti-Iba1 | Abcam | ab5076 | 1:500 |
| Anti-NeuN | Abcam | Ab104224 | 1:1000 |
| Donkey Anti-Goat IgG H&L (FITC) preadsorbed ab7121 | Abcam | ab7127 | 1:200 |
| Dylight594-AffiniPure Donkey Anti-Mouse IgG (H+L) | BOSTER Biological Technology co. ltd | BA1148 | 1:200 |
| TRITC Conjugated AffiniPure Goat Anti-rabbit IgG (H+L) | BOSTER Biological Technology co. ltd | BA1090 | 1:200 |

**Supplementary Table S5: Distribution of animals according to different groups and mortality rate.**

| **Groups** | **Mortality** | **Exclude** |
| --- | --- | --- |
| **Experiment 1** |  |  |
| Sham | 0（0/12） | 0 |
| SAH（6h,12h,24h,72h） | 22.7%(10/44) | 4 |
| **Experiment 2** |  |  |
| Sham | 0(0/12) | 0 |
| SAH+Vehicle | 17.6%(3/17) | 2 |
| SAH+RU.521(150µg/kg) | 23.5%(4/17) | 1 |
| SAH+RU.521(450µg/kg) | 14.3%(2/14) | 0 |
| SAH+RU.521(1350µg/kg) | 18.8%(3/16) | 1 |
| **Experiment 3** |  |  |
| Sham | 0(0/12) | 0 |
| SAH+Vehicle | 18.8%(3/16) | 1 |
| SAH+RU.521 | 22.2%(4/18) | 2 |
| **Experiment 4** |  |  |
| Sham | 0(0/18) | 0 |
| SAH+Vehicle | 26.9%(7/26) | 1 |
| SAH+RU.521 | 20%(5/25) | 2 |
| SAH+PBS+RU.521 | 20.8%(5/24) | 1 |
| SAH+2’3’-cGAMP+RU.521 | 23.1%(6/26) | 2 |
| **Total** |  |  |
| Sham | 0(0/54) | 0 |
| SAH | 21.4%(52/243) | 17 |

SAH, subarachnoid hemorrhage; RU.521, selective small molecule inhibitor for cGAS; Vehicle, 1% DMSO + corn oil; 2’3’-cGAMP, a second messenger converted by activated cGAS; PBS, phosphate-buffered saline; Oxy-Hb, oxyhemoglobin.
