# Supplementary material for: RU.521 mitigates subarachnoid hemorrhage-induced brain injury via regulating microglial polarization and neuroinflammation mediated by the cGAS/STING/NF-κB pathway
Source: Cell Commun Signal. 2023 Sep 28;21:264. doi: 10.1186/s12964-023-01274-2 (PMC10537158; doi:10.1186/s12964-023-01274-2)

Additional file: Uncropped Western bot gel images. Boxes highlight lanes used in figures.

Figure 1A

cGAS 60KD

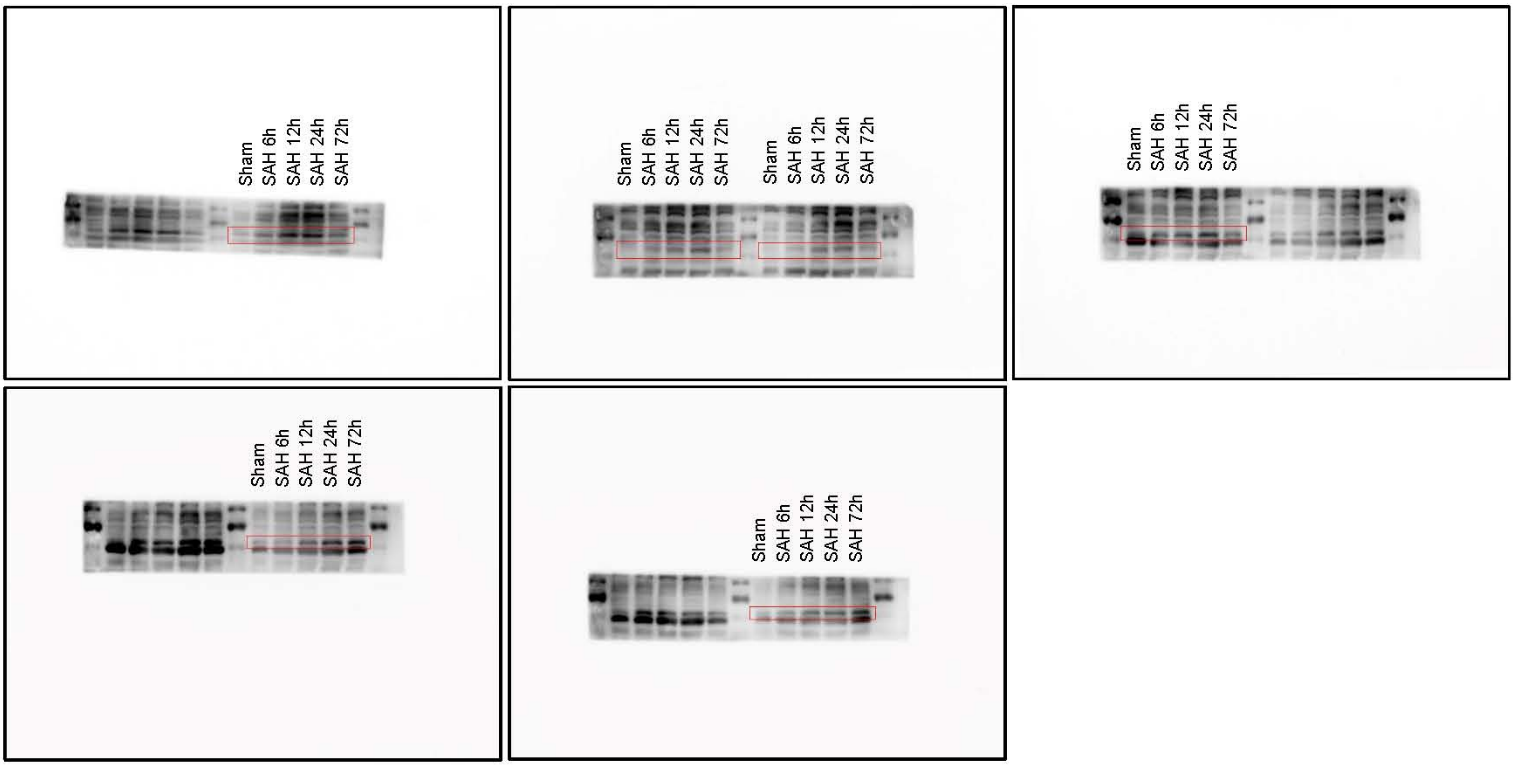

β-tubulin 55KD

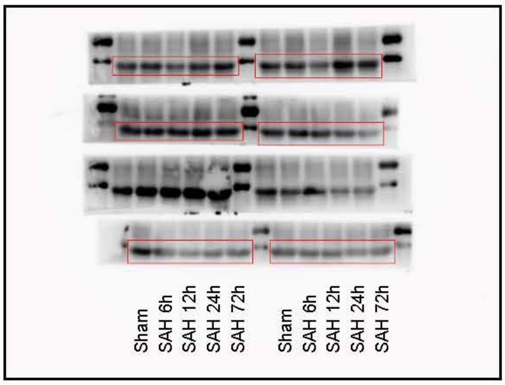

Figure 3A

Iba1 17KD

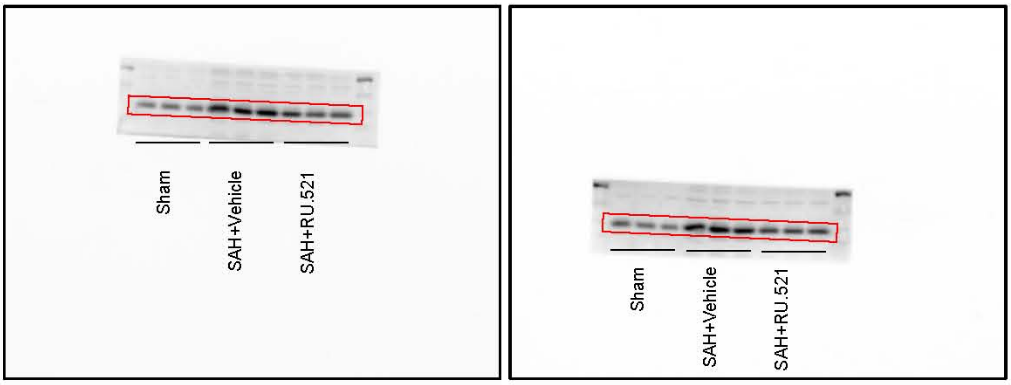

β-tubulin 55KD

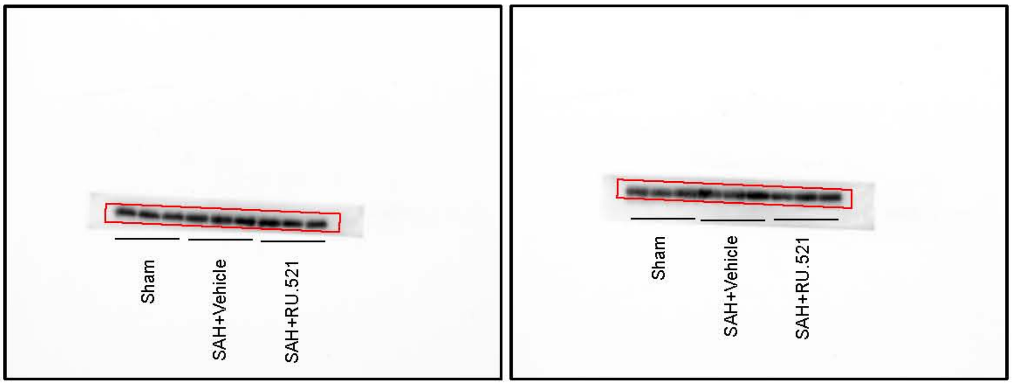

Figure 4A

p-STING 42KD

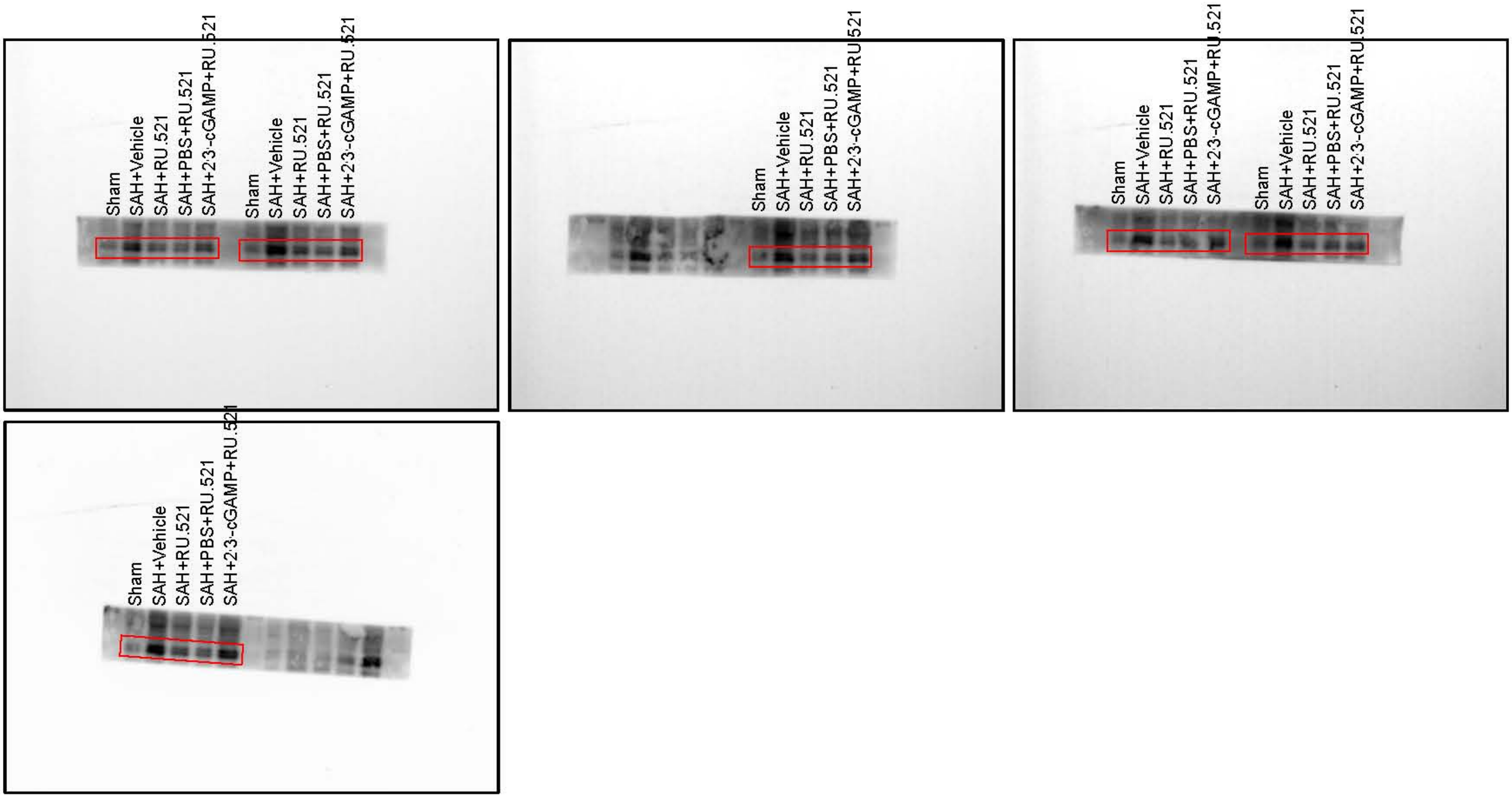

STING 42KD

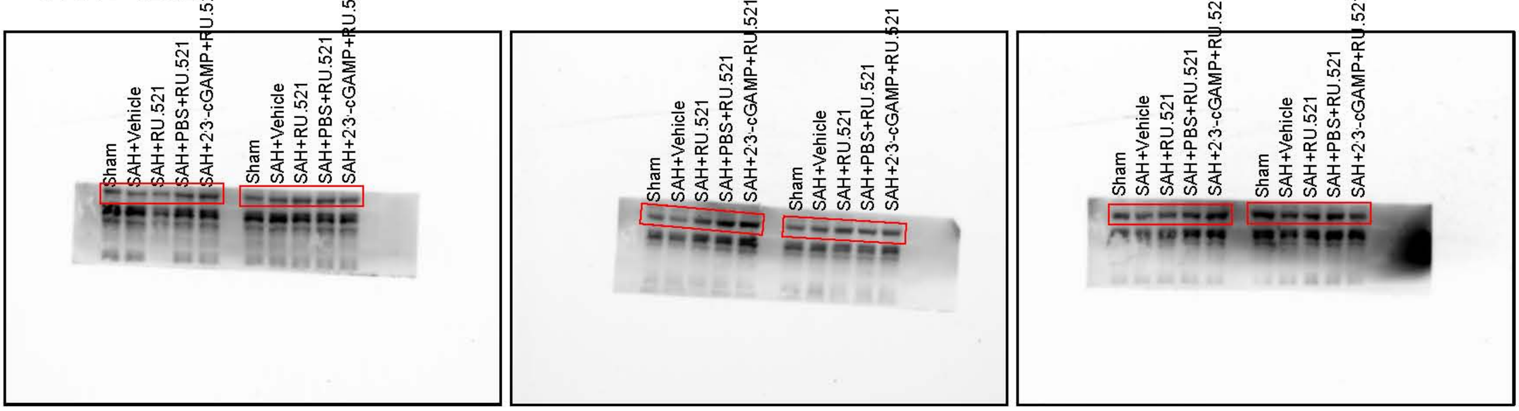

p-TBK1 84KD

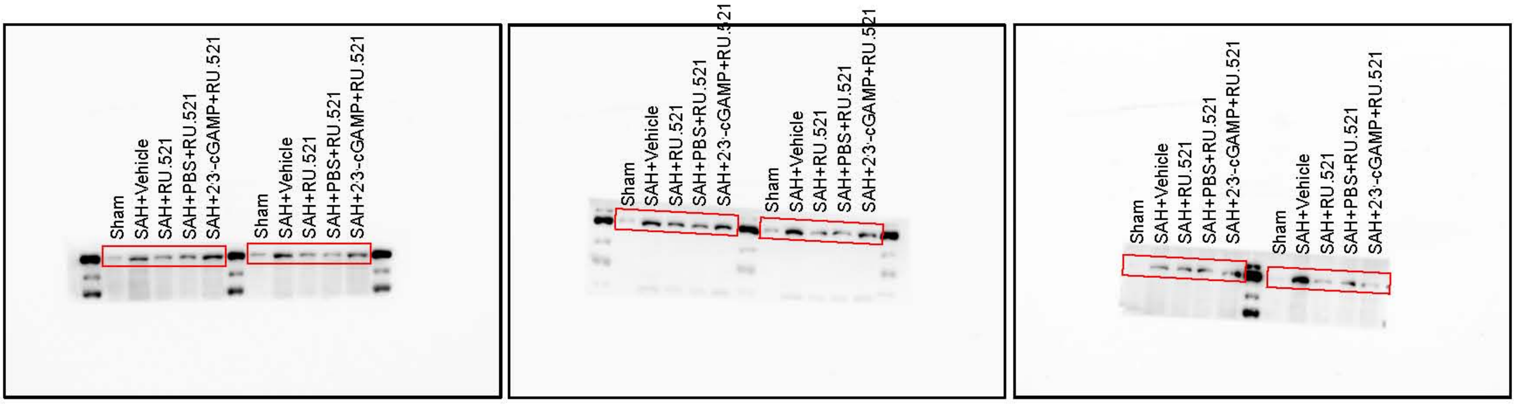

TBK1 84KD

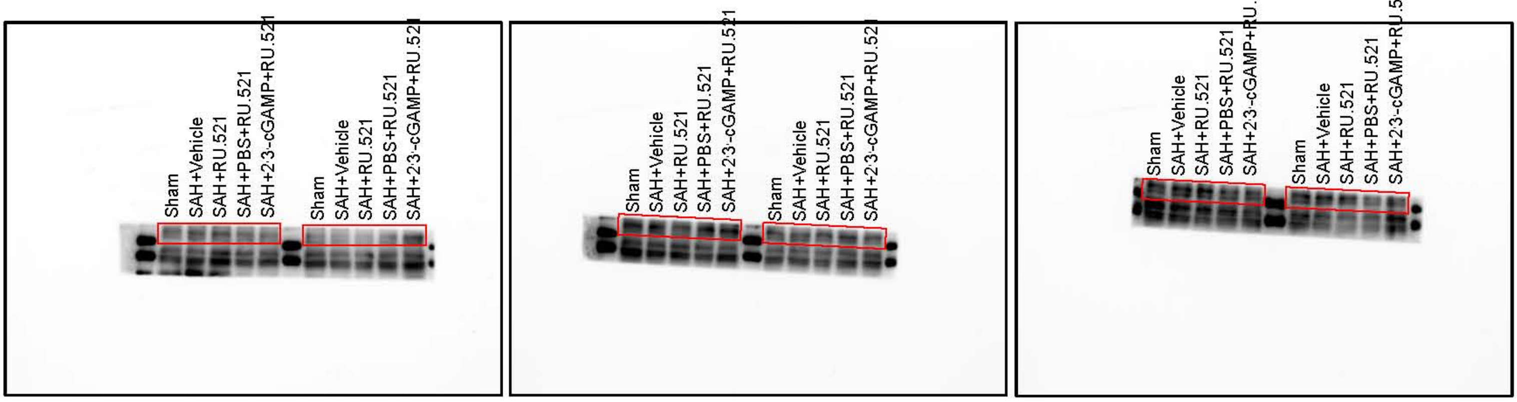

p-NF-κB p65 65KD

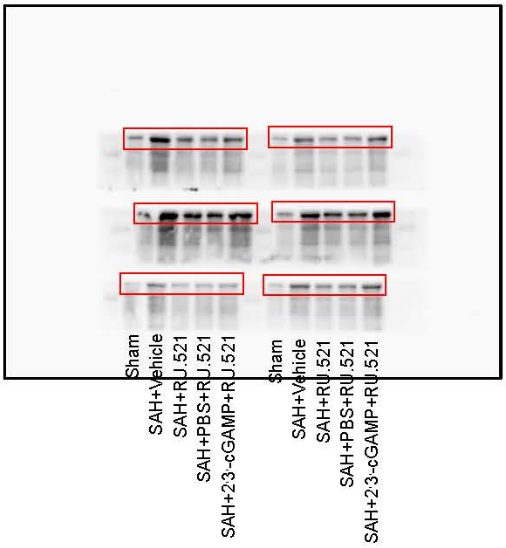

NF-κB p65 65KD

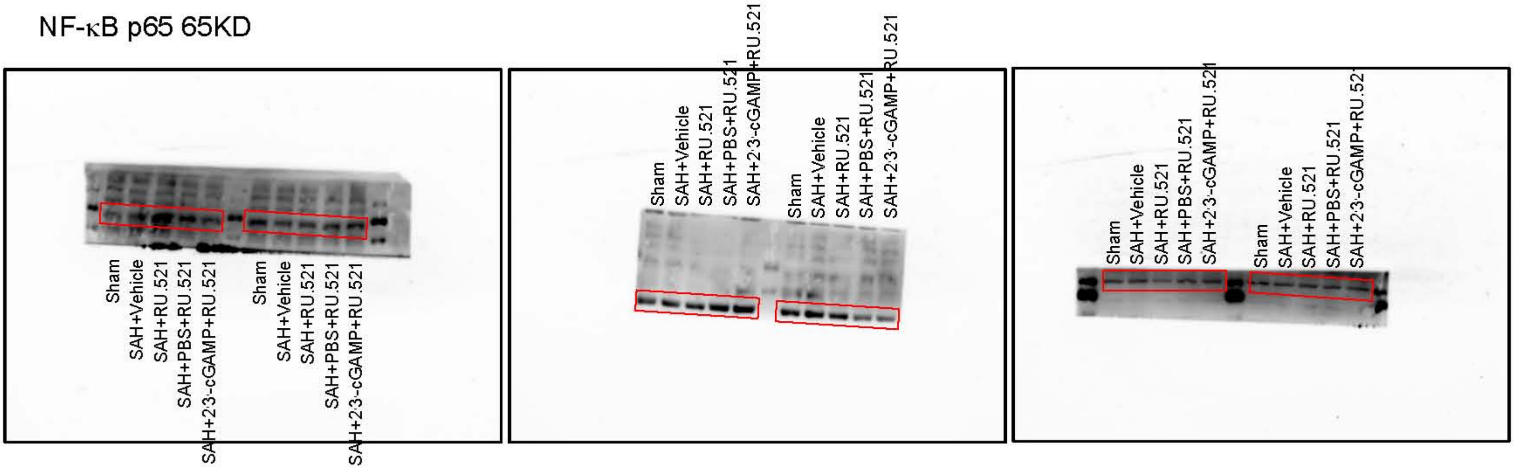

Arg-1 37KD

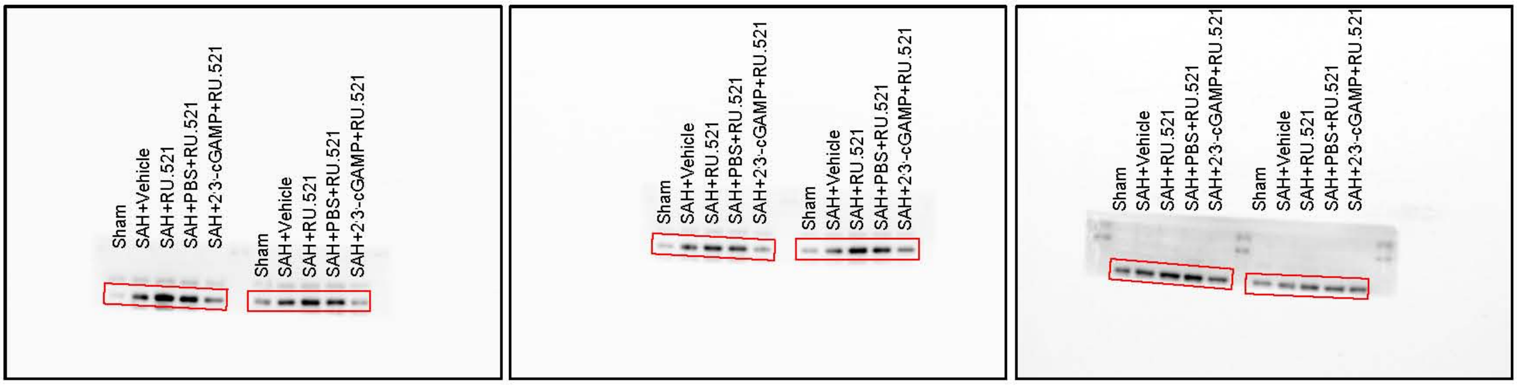

CD16 42KD

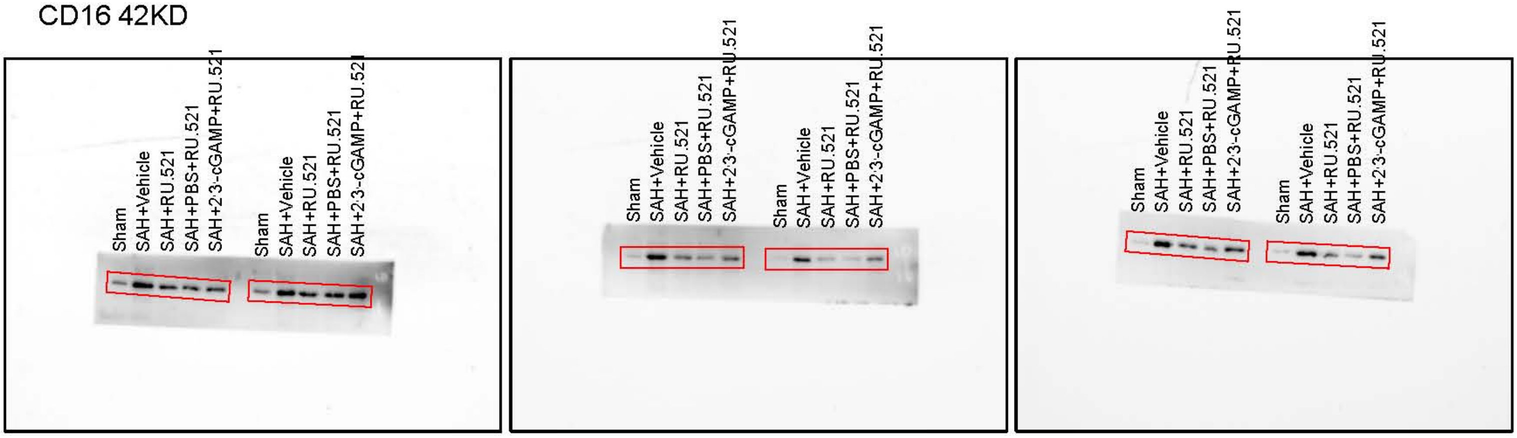

IL-10 18KD

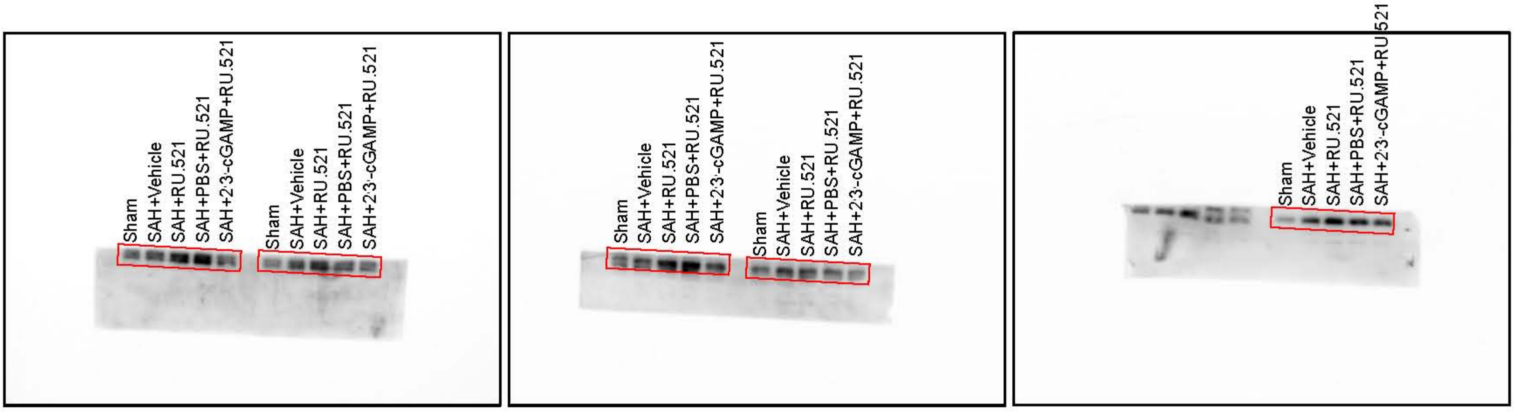

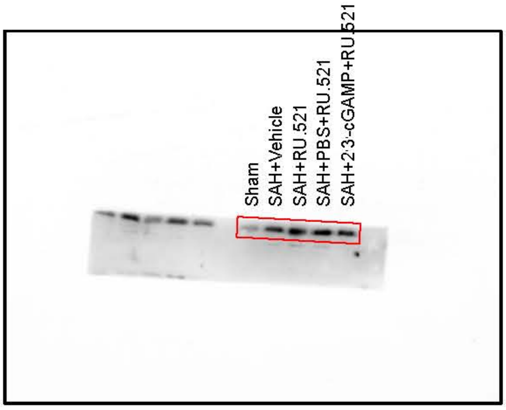

IL-6 23KD

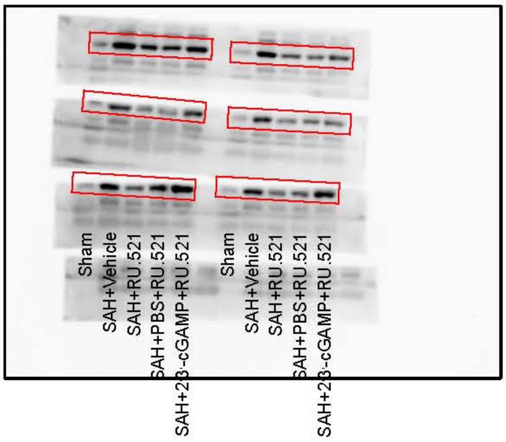

TNF-α 26KD

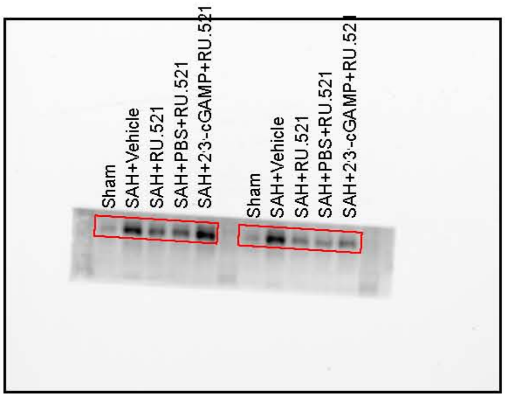

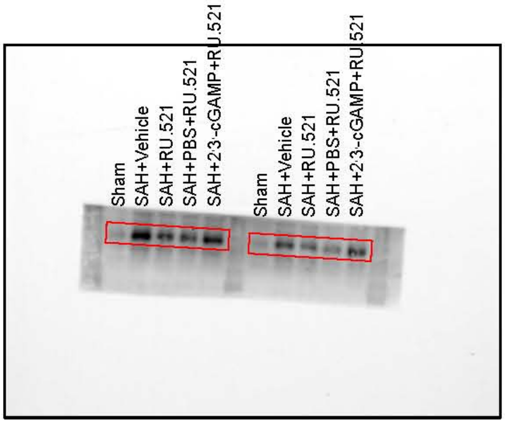

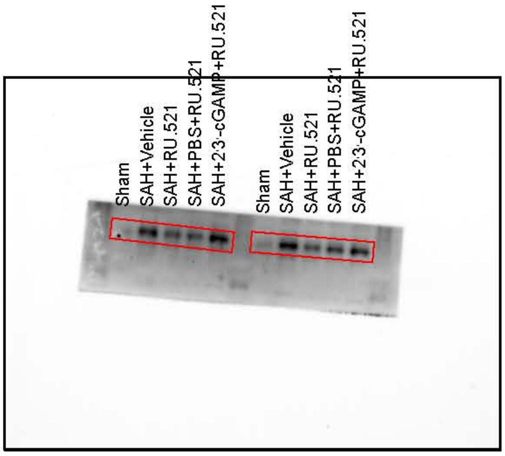

IL-1β 31KD

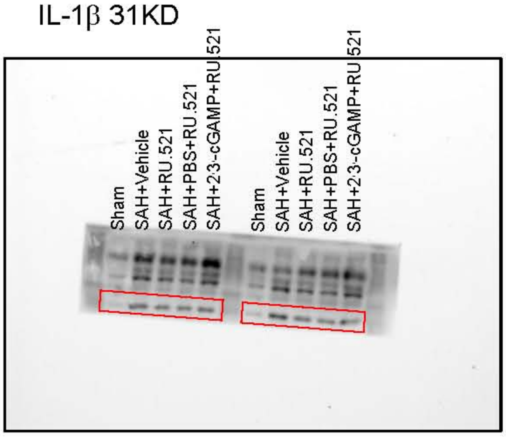

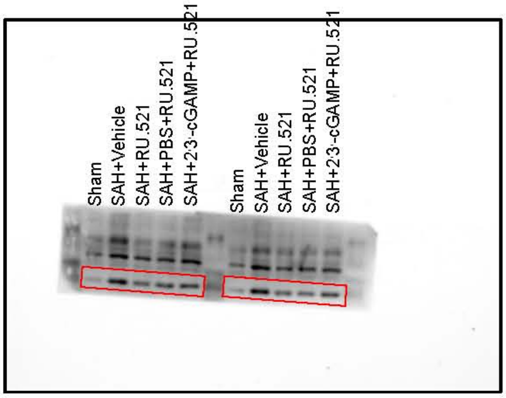

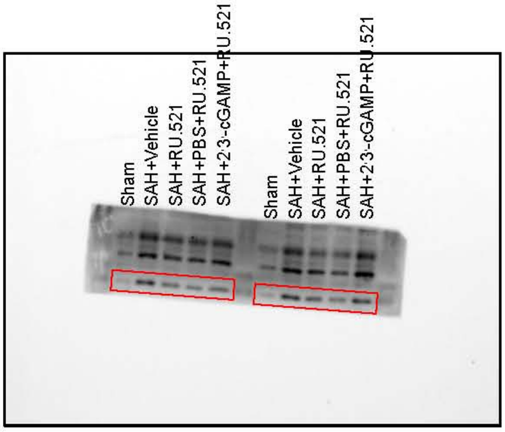

β-tubulin 55KD

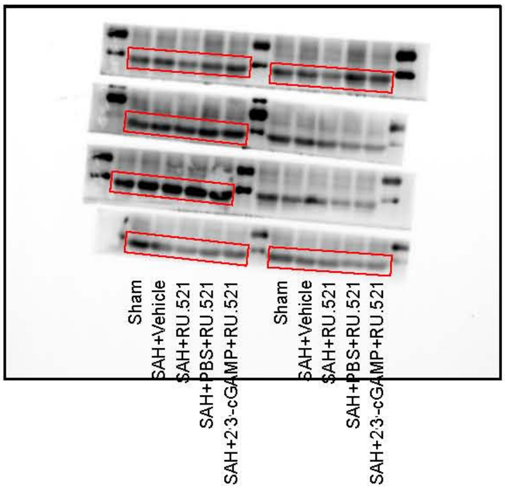

Figure 6B

Cleaved caspase-3 19KD

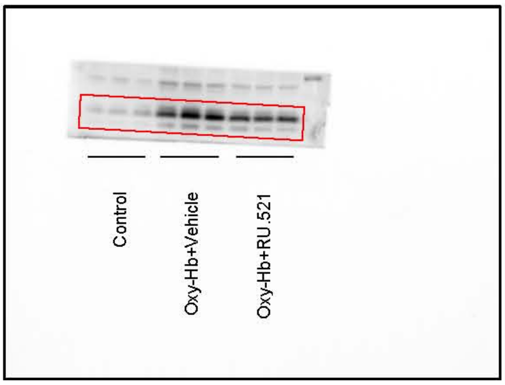

β-tubulin 55KD

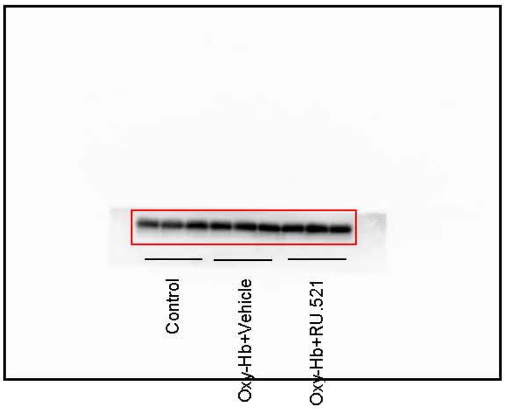

Figure 7A

Arg-1 37KD

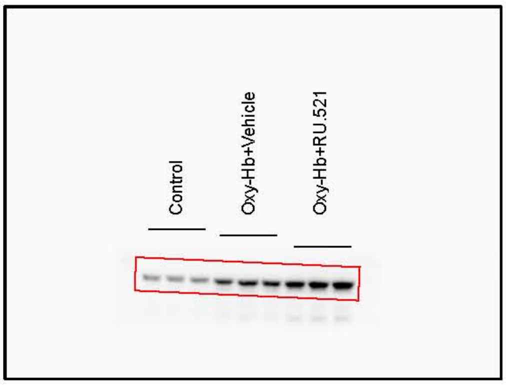

CD16 42KD

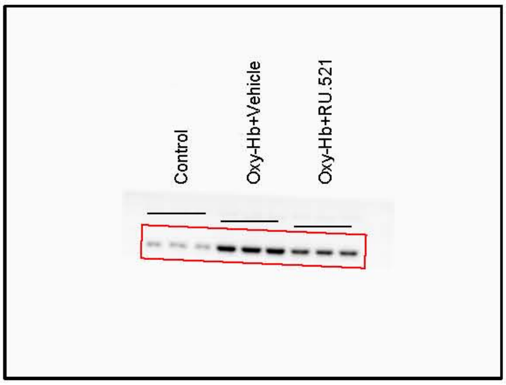

iNOS 130KD

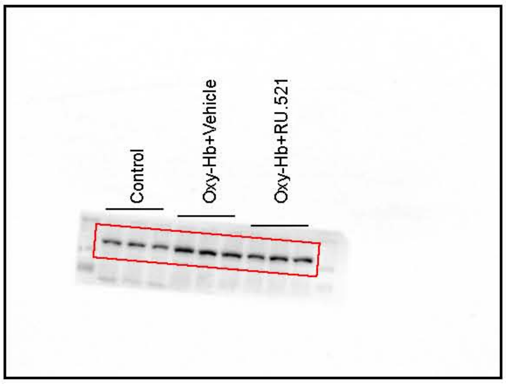

CD206 166KD

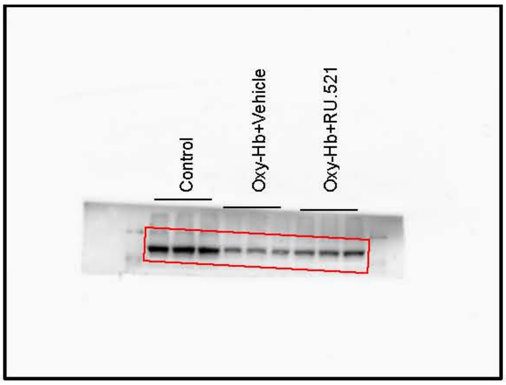

IL-10 18KD

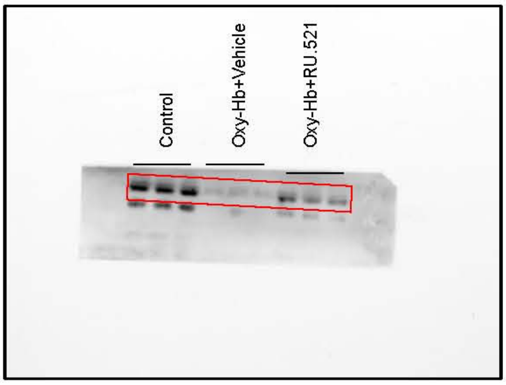

IL-6 23KD

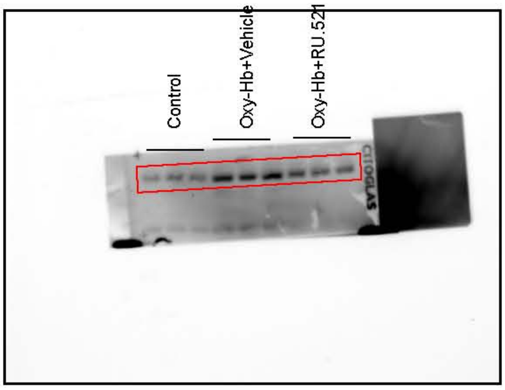

TNF-α 26KD

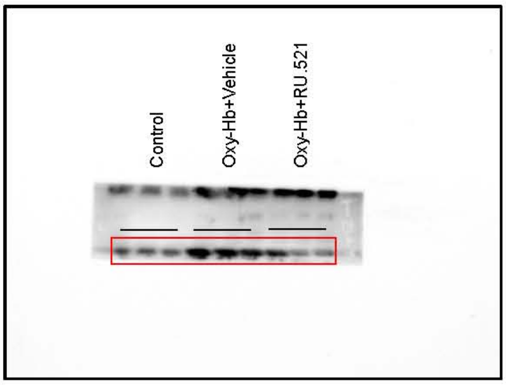

IL-1β 31KD

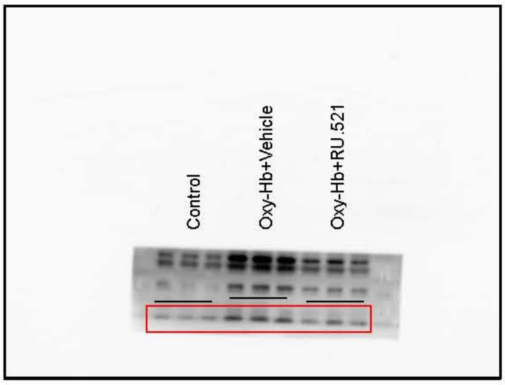

β-tubulin 55KD

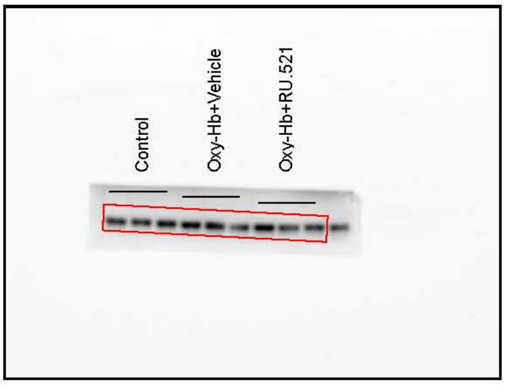

Figure 8A

cGAS 60KD

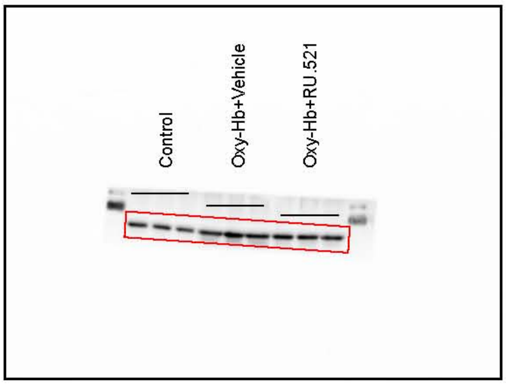

p-STING 42KD

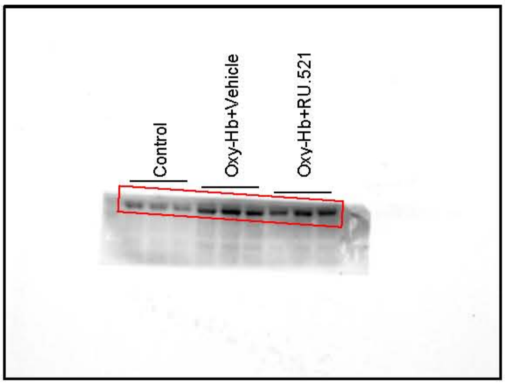

STING 42KD

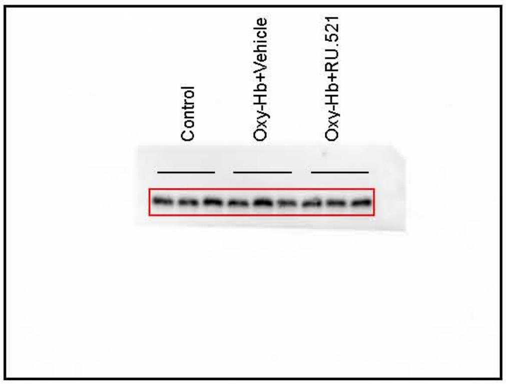

p-TBK1 84KD

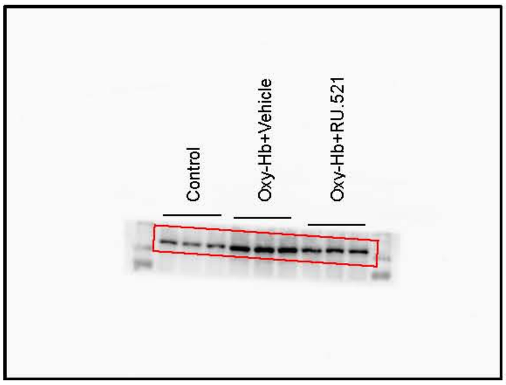

TBK1 84KD

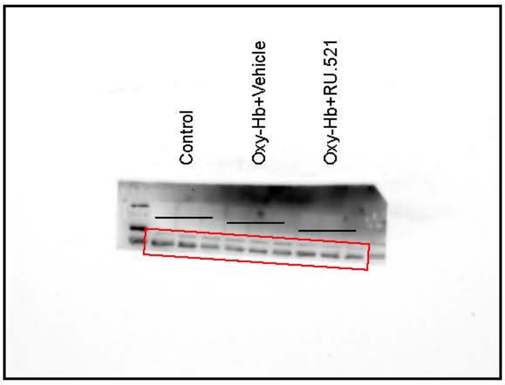

β-tubulin 55KD

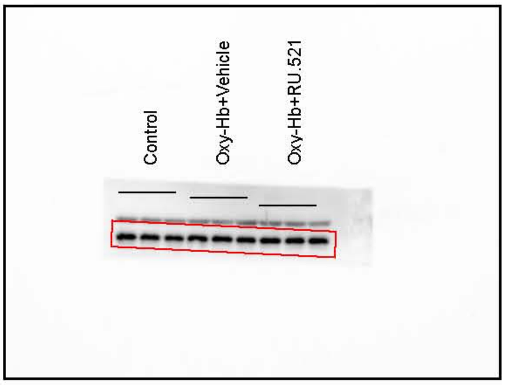

Figure 8C

whole lysate

p-IKBα 39KD

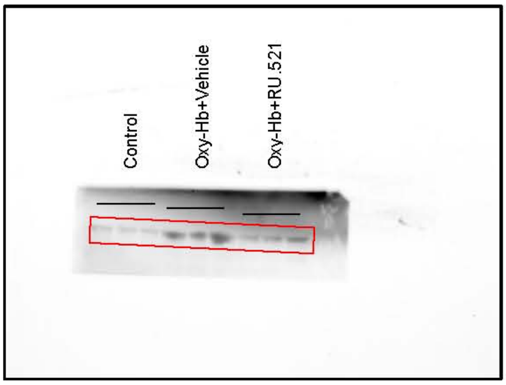

IKBα 39KD

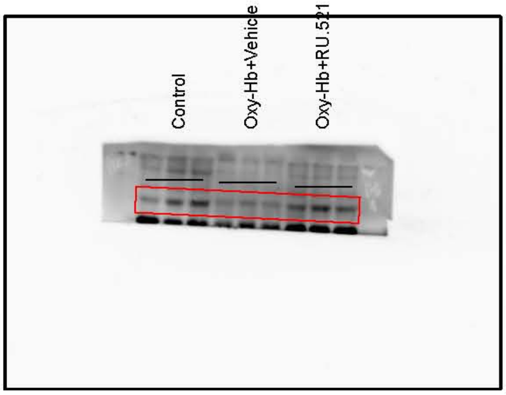

β-actin 42KD

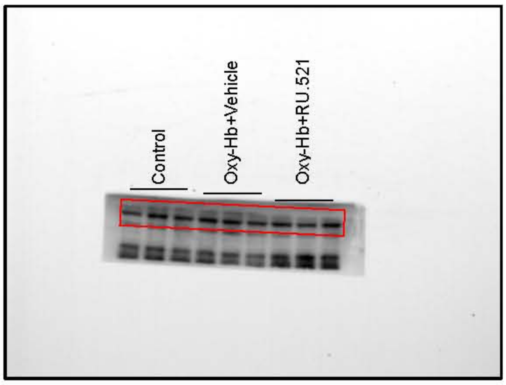

cytoplasm

NF-κB p65 65KD

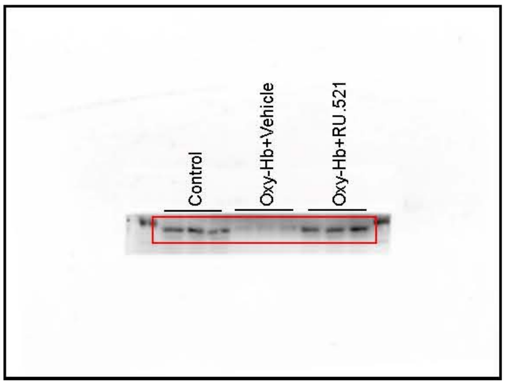

β-actin 42KD

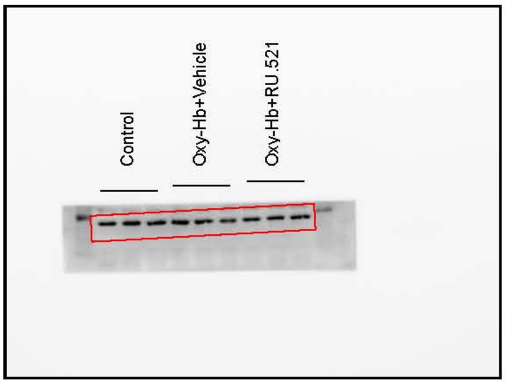

Lamin B1 66KD

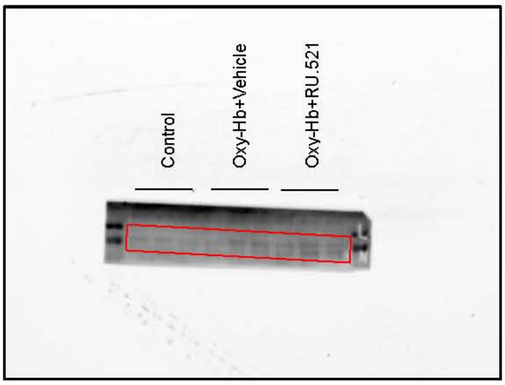

nuclear

NF-κB p65 65KD

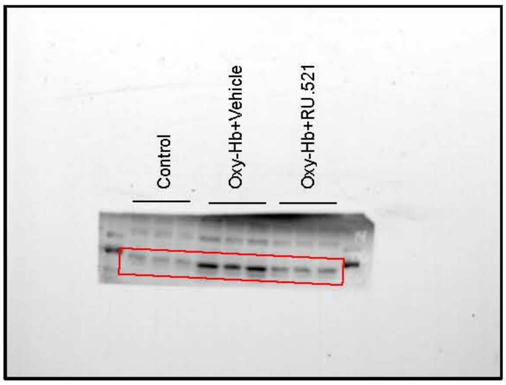

β-actin 42KD

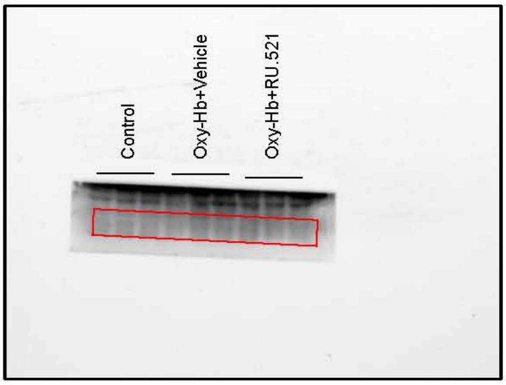

Lamin B1 66KD

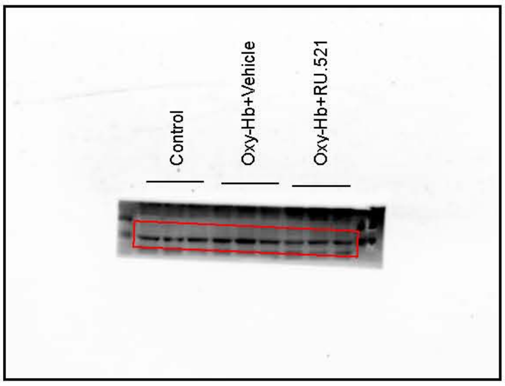

Supplement: Supplementary file 3 — Additional file 2. Uncropped Western bot gel images. [file 12964_2023_1274_MOESM2_ESM.pdf]
