# Supplementary material for: Correction: RU.521 mitigates subarachnoid hemorrhage-induced brain injury via regulating microglial polarization and neuroinflammation mediated by the cGAS/STING/NF-κB pathway
Source: Cell Commun Signal. 2024 Aug 6;22:390. doi: 10.1186/s12964-024-01772-x (PMC11301847; doi:10.1186/s12964-024-01772-x)

Additional file: Uncropped Western bot gel images. Boxes highlight lanes used in figures.

Figure 1A

cGAS 60KD

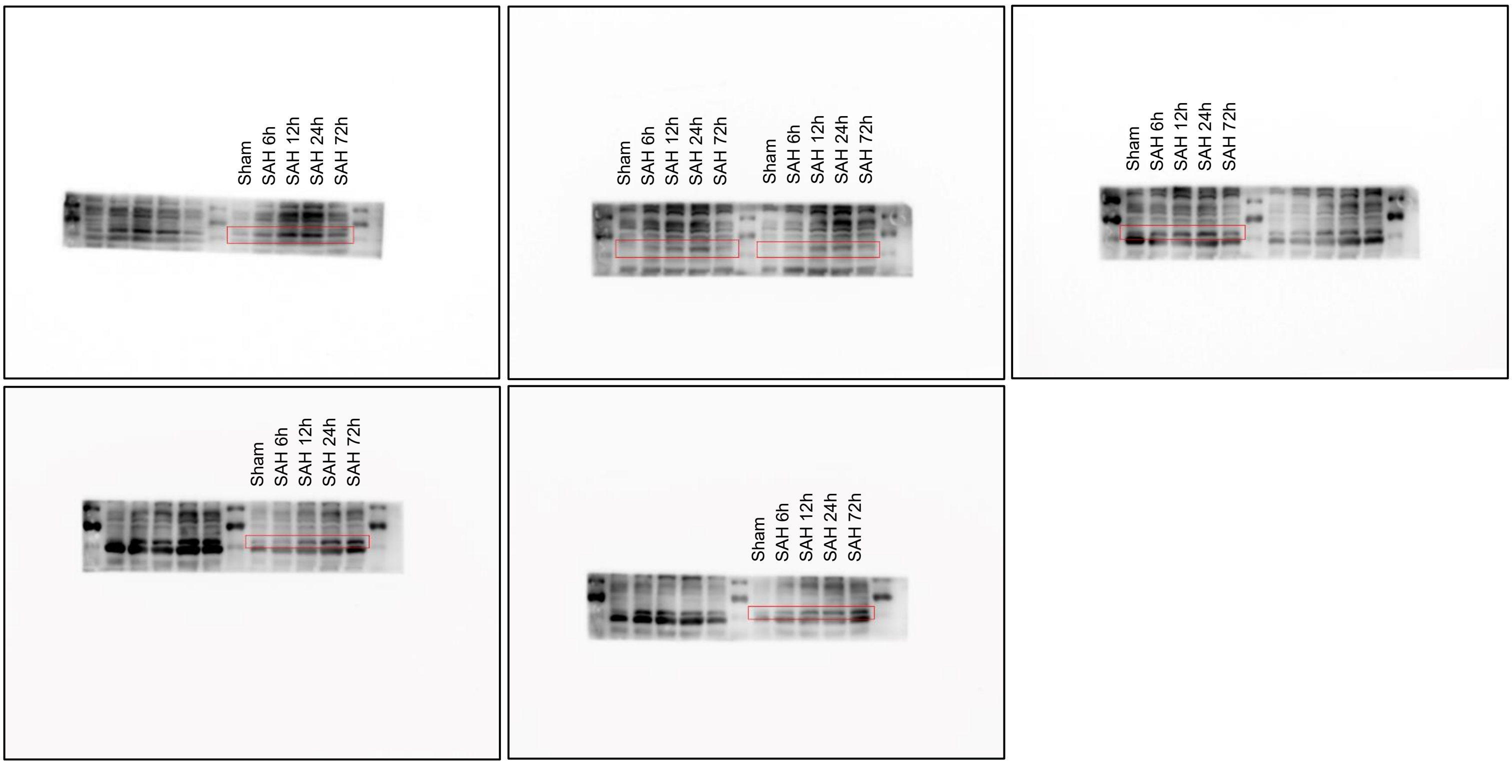

β-tubulin 55KD

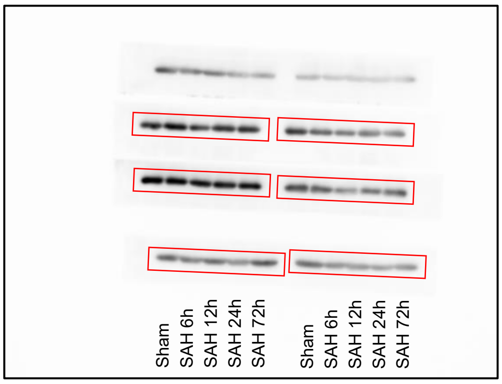

Figure 3A

Iba1 17KD

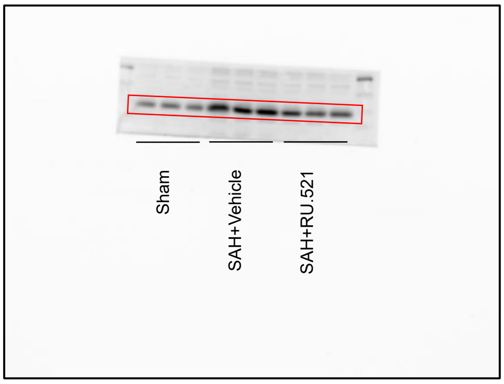

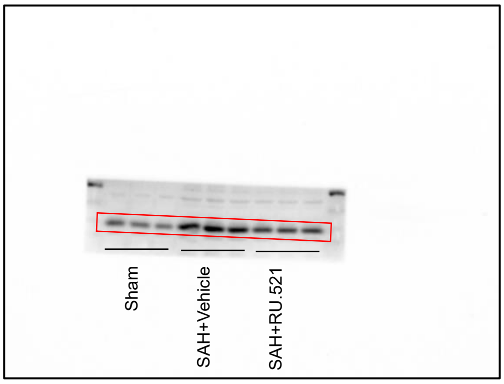

β-tubulin 55KD

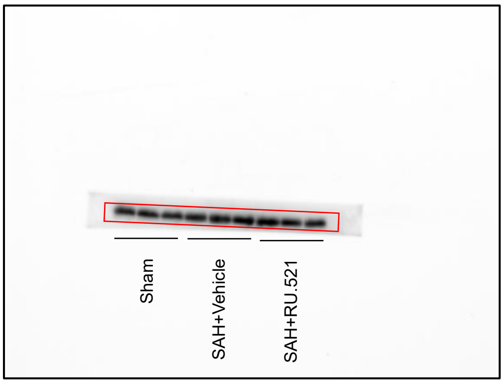

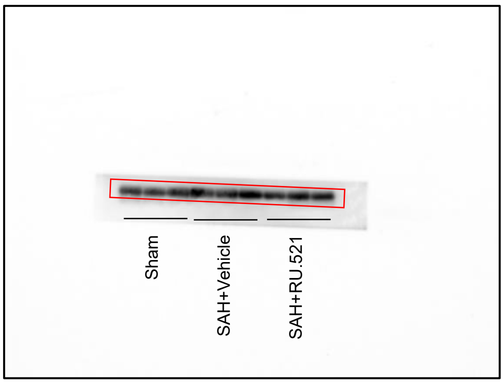

Figure 4A

p-STING 42KD

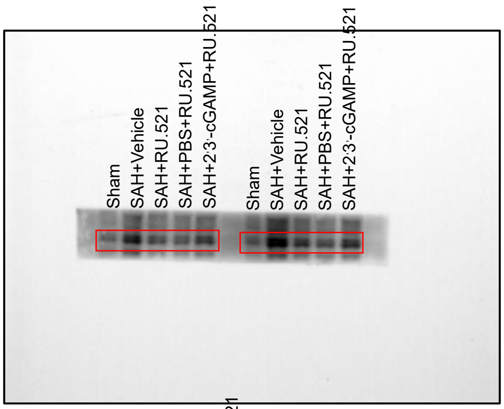

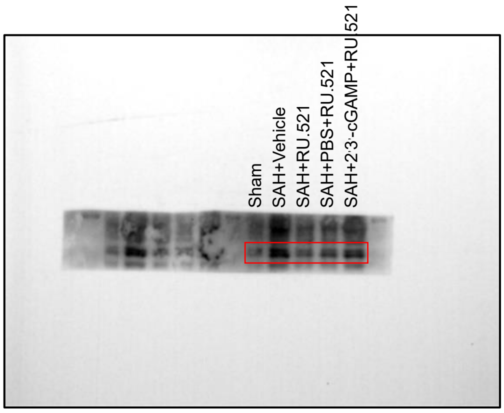

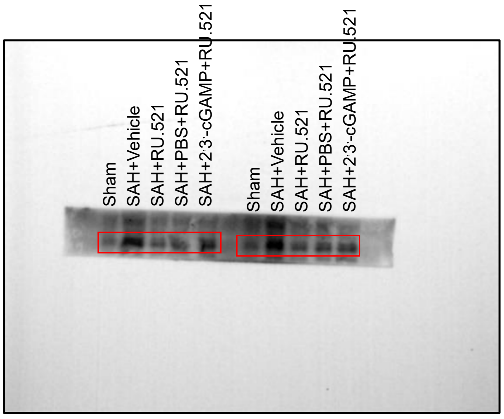

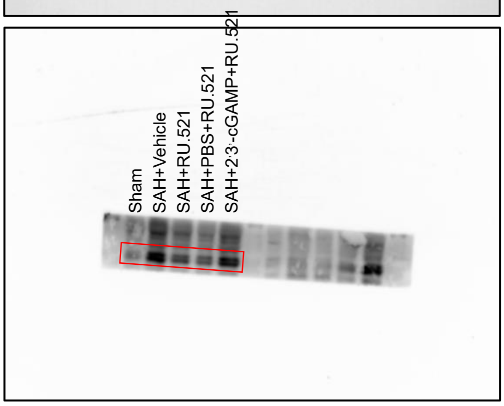

STING 42KD

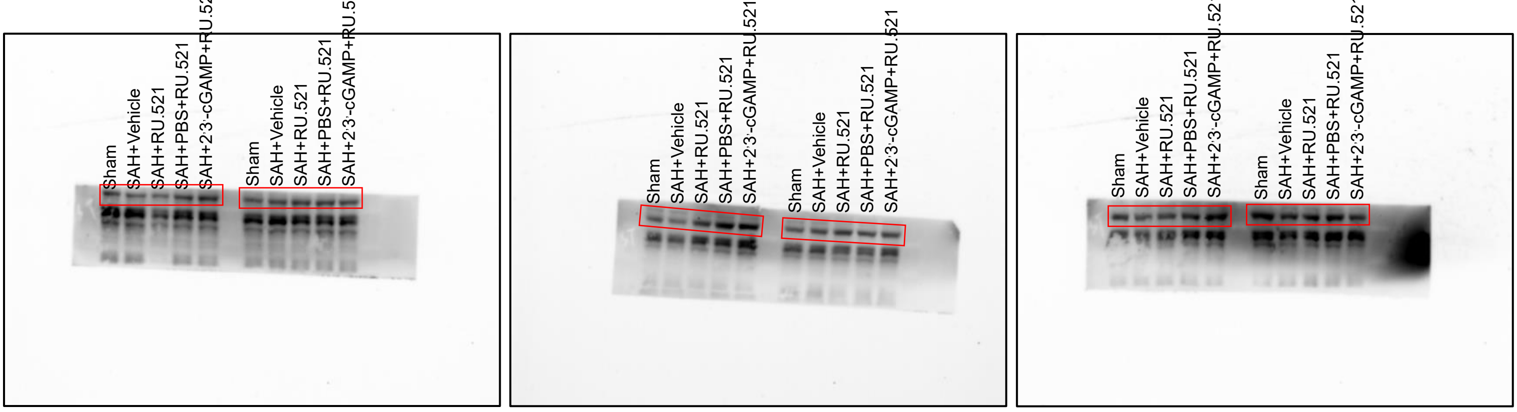

p-TBK1 84KD

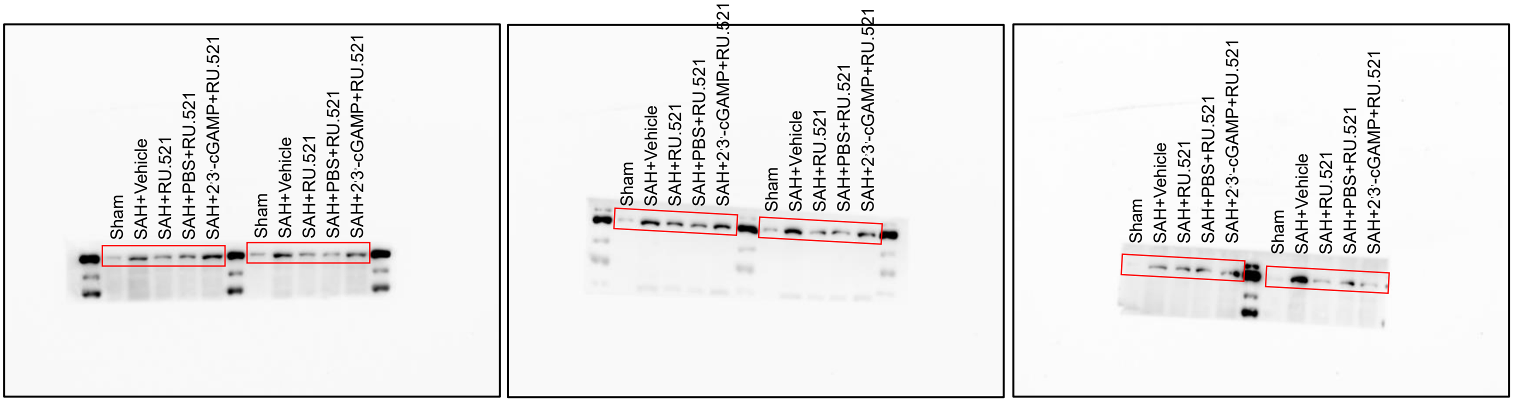

TBK1 84KD

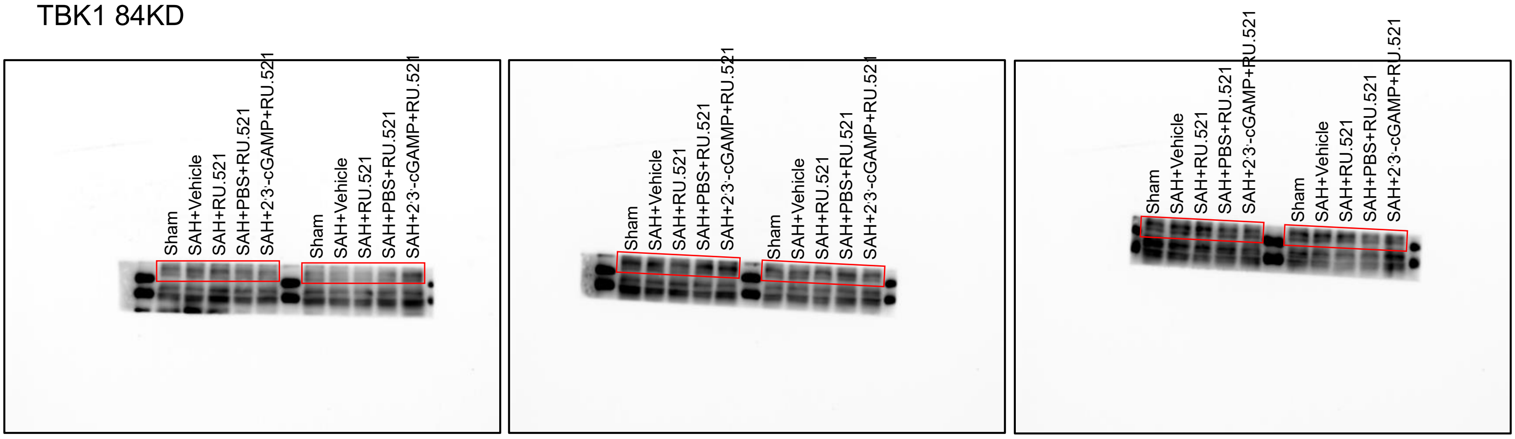

p-NF-κB p65 65KD

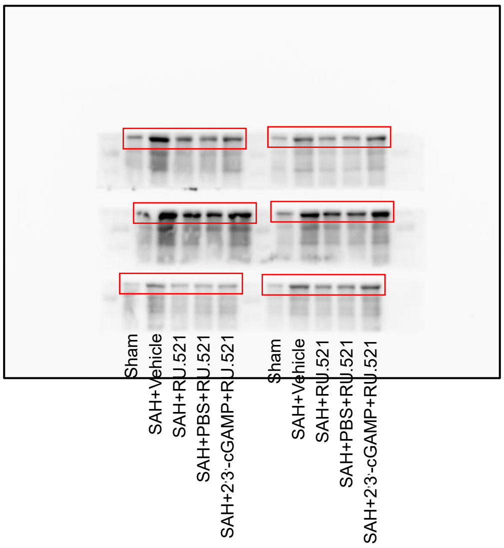

NF-κB p65 65KD

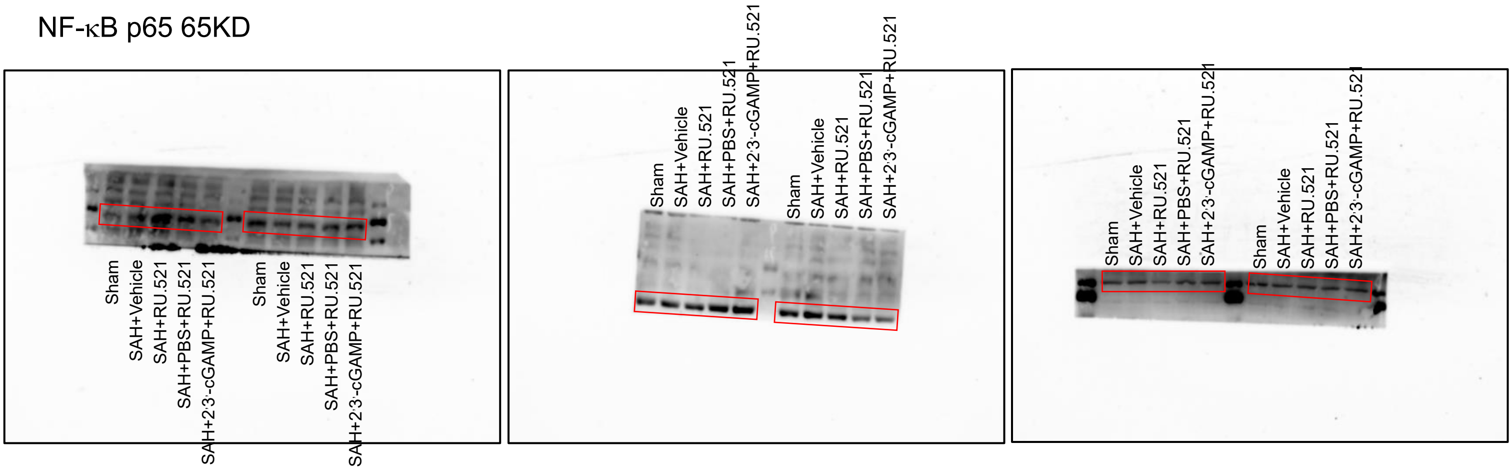

Arg-1 37KD

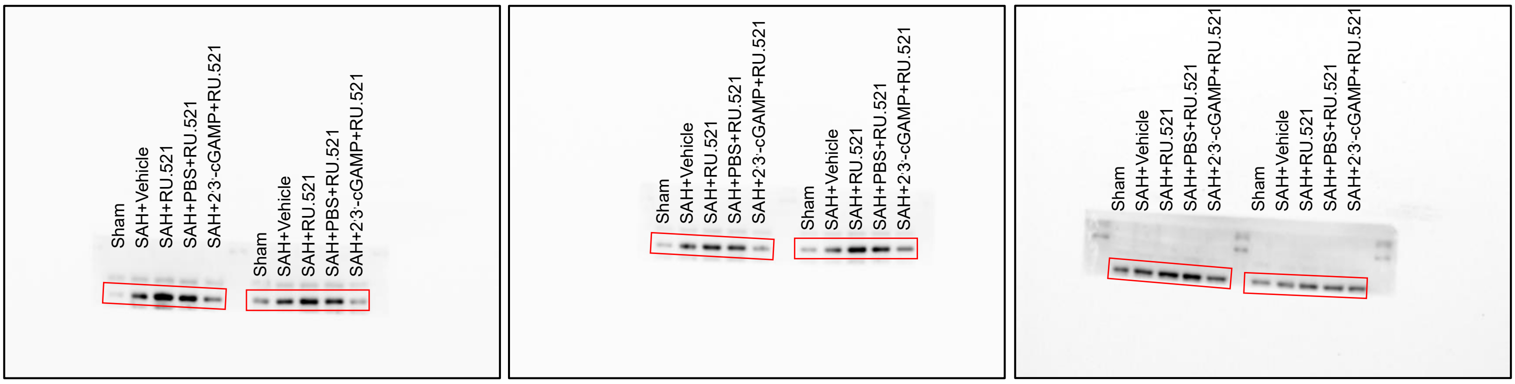

CD16 42KD

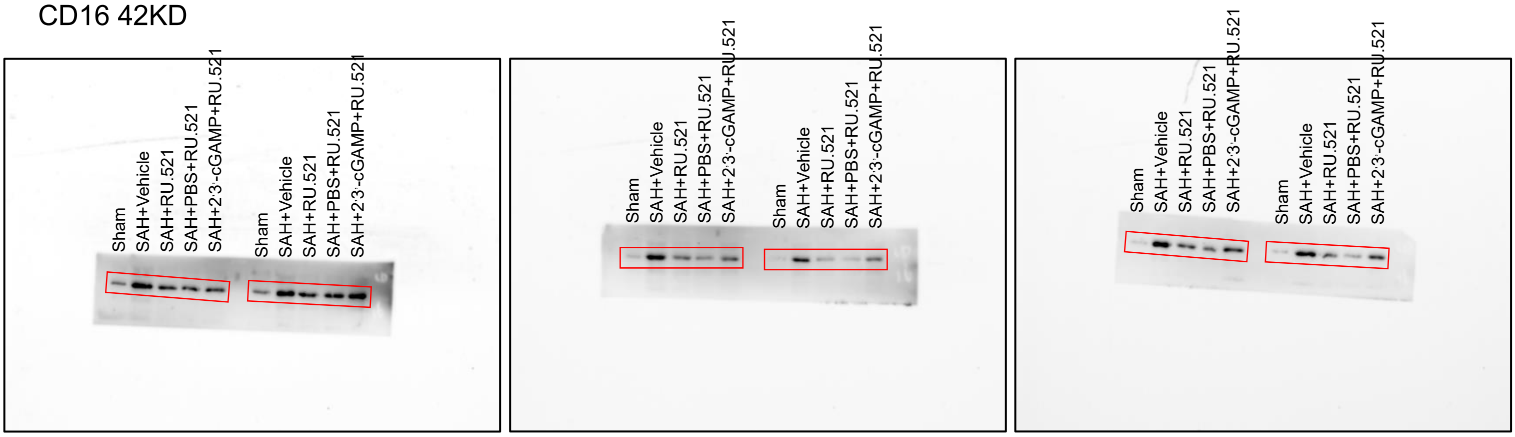

IL-10 18KD

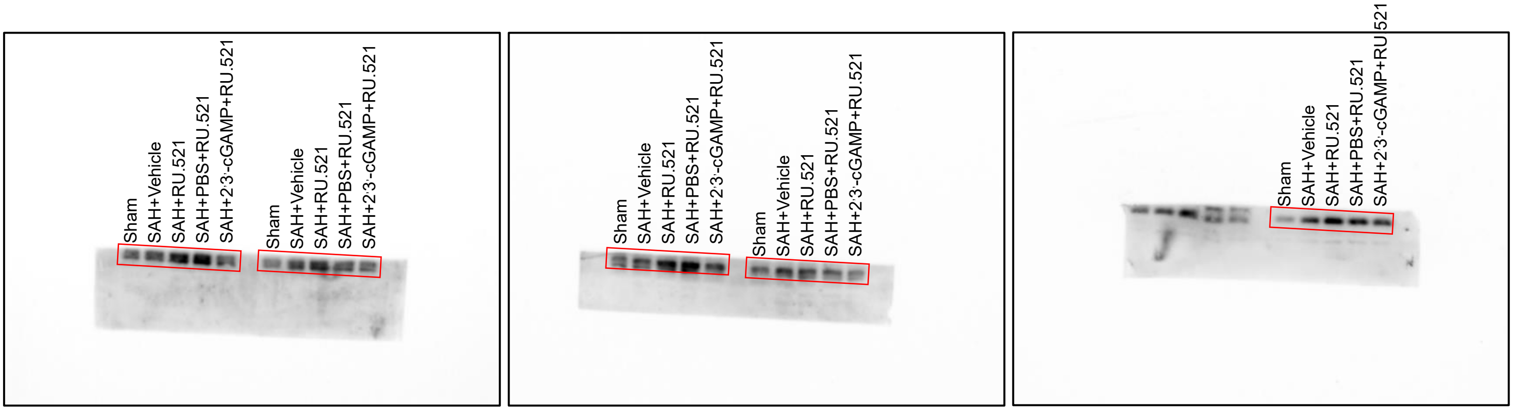

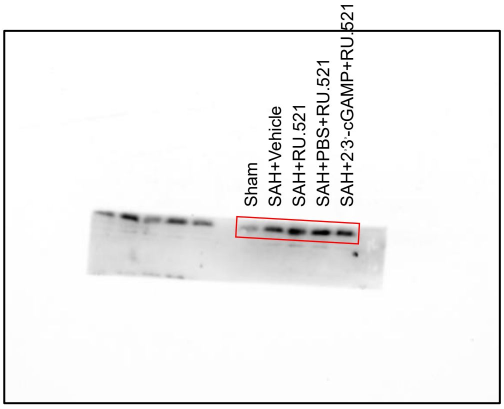

IL-6 23KD

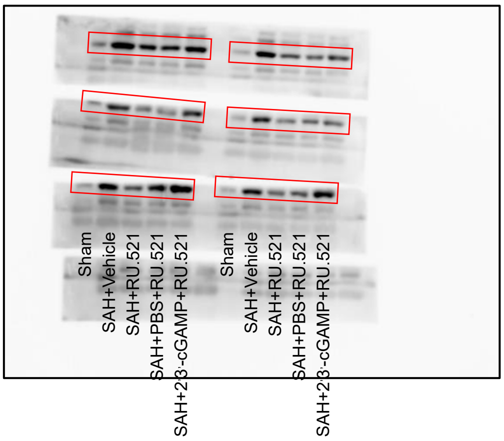

TNF-α 26KD

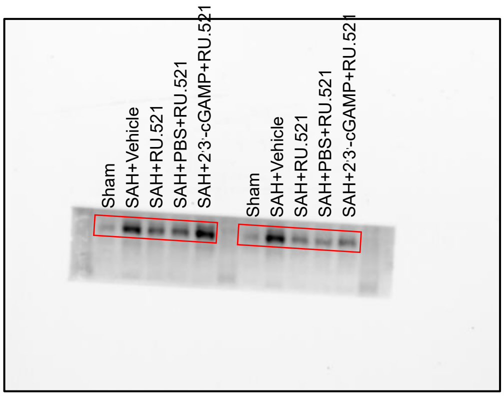

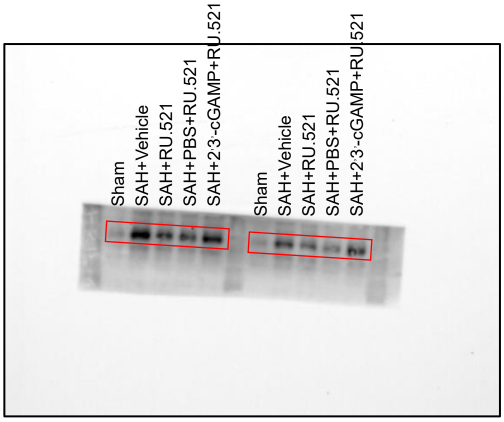

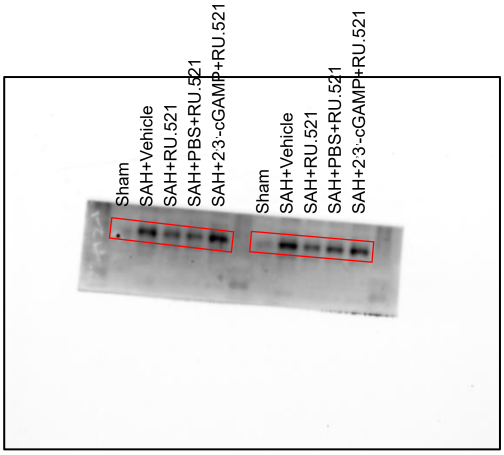

IL-1β 31KD

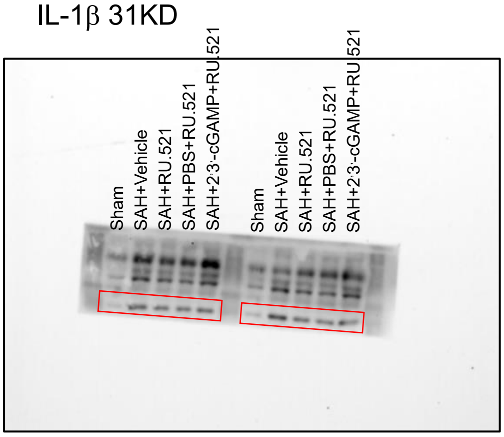

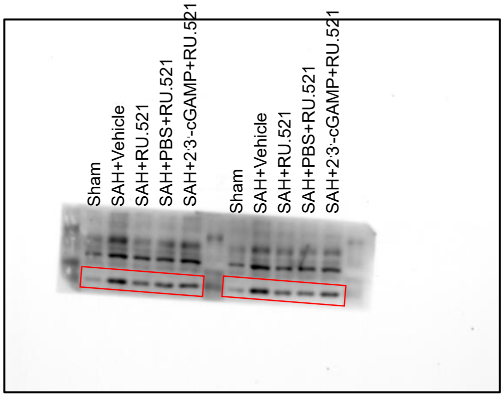

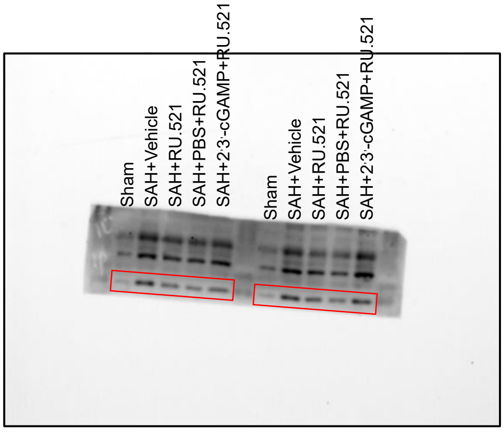

β-tubulin 55KD

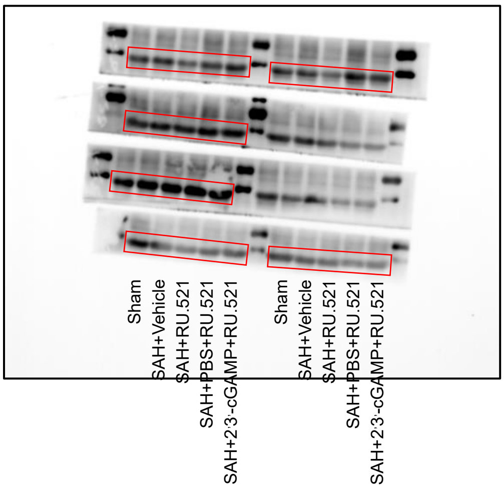

Figure 6B

Cleaved caspase-3 19KD

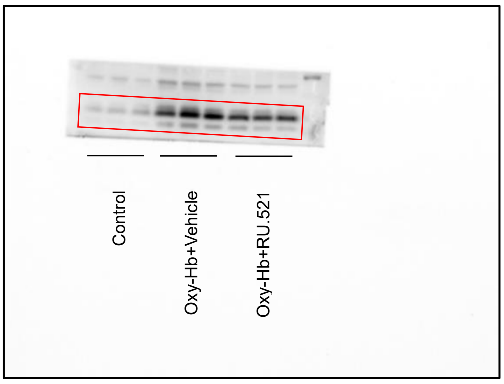

β-tubulin 55KD

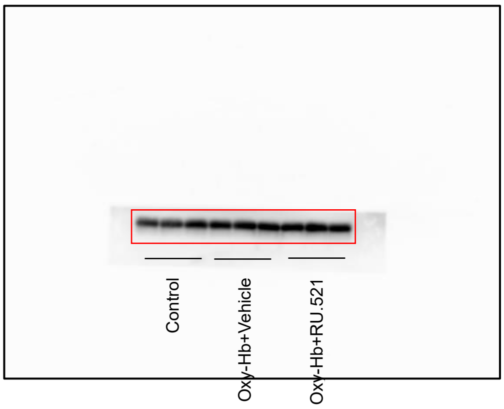

Figure 7A

Arg-1 37KD

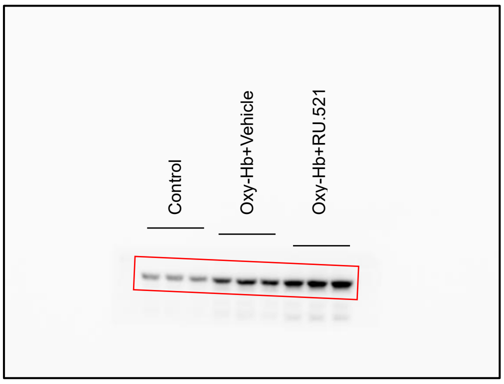

CD16 42KD

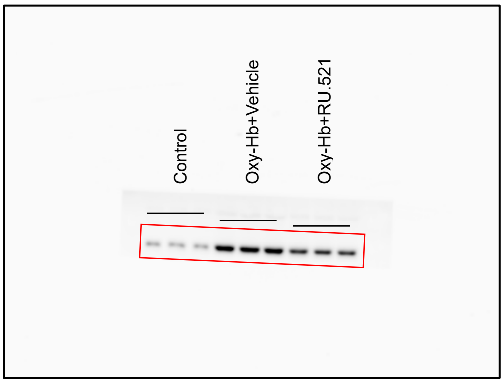

iNOS 130KD

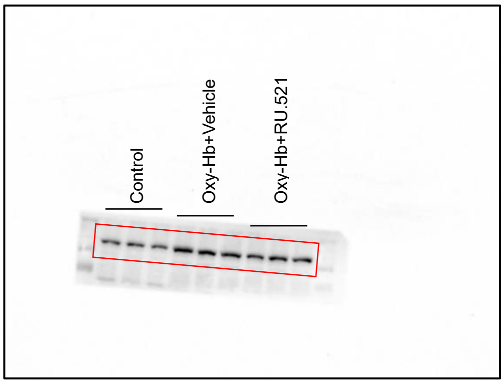

CD206 166KD

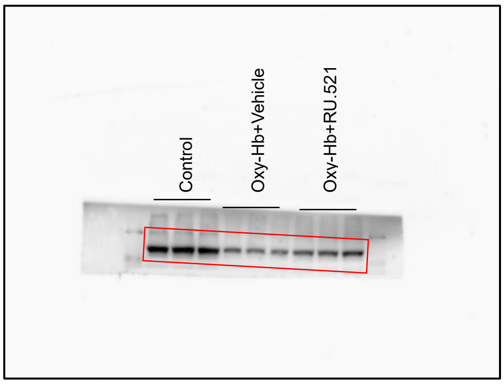

IL-10 18KD

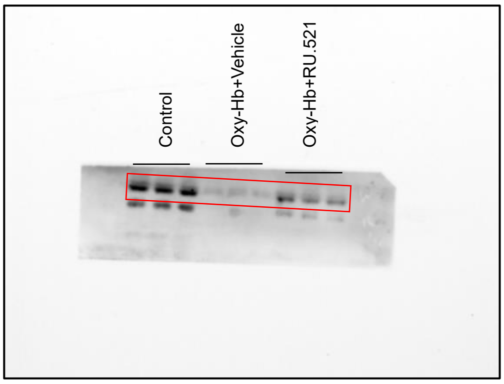

IL-6 23KD

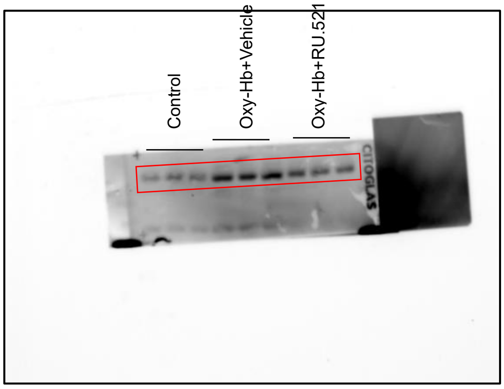

TNF-α 26KD

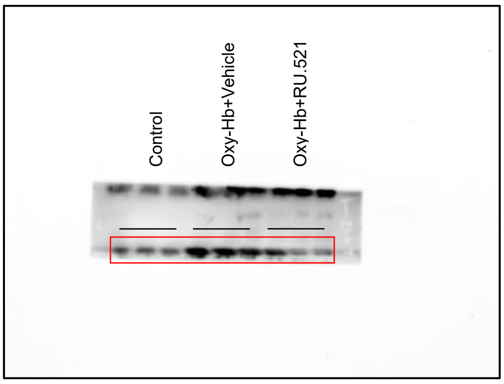

IL-1β 31KD

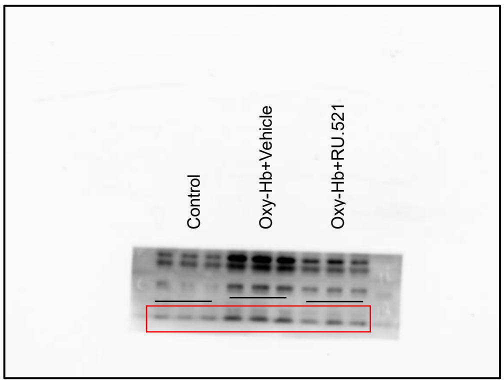

β-tubulin 55KD

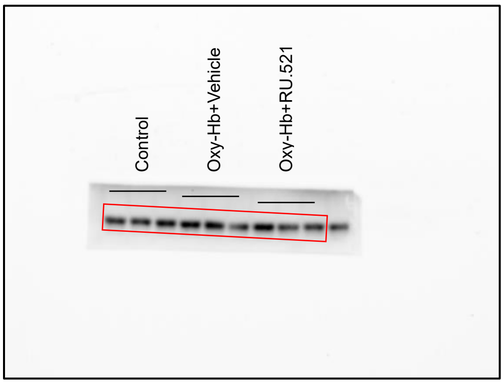

Figure 8A

cGAS 60KD

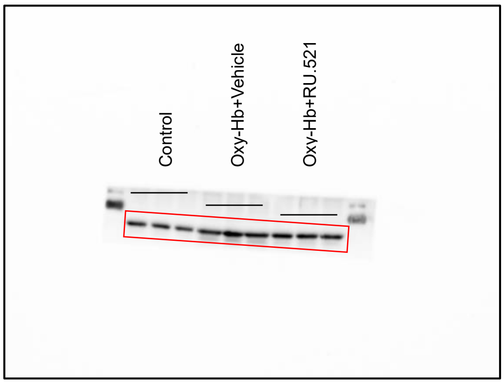

p-STING 42KD

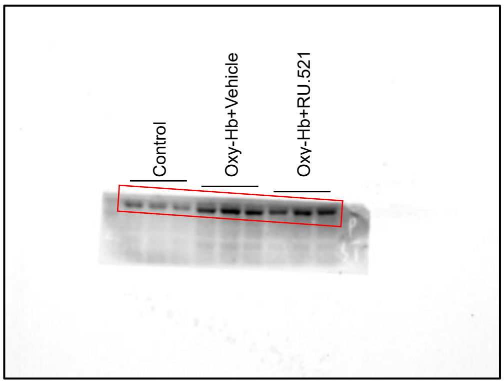

STING 42KD

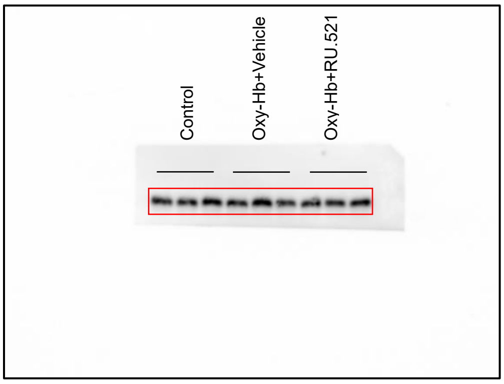

p-TBK1 84KD

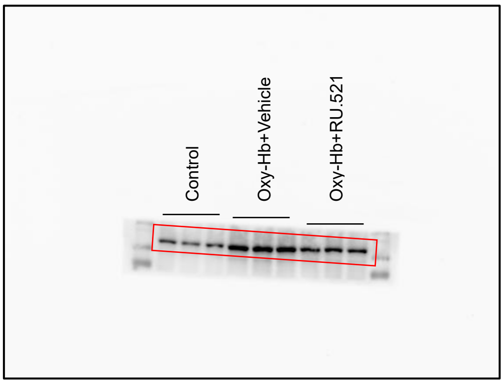

TBK1 84KD

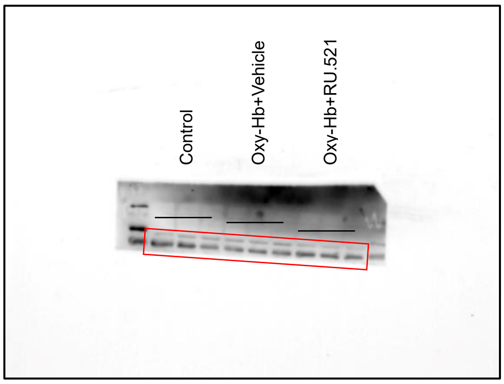

β-tubulin 55KD

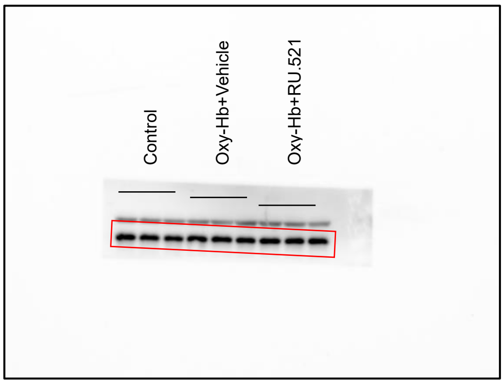

Figure 8C

whole lysate

p-IKBα 39KD

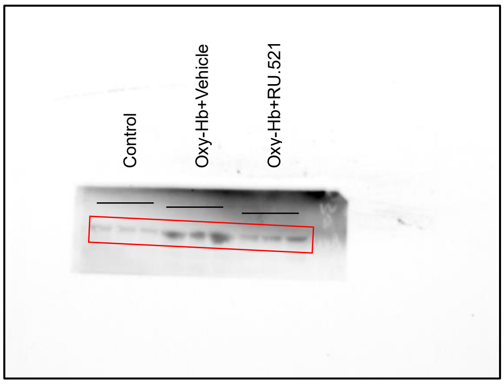

IKBα 39KD

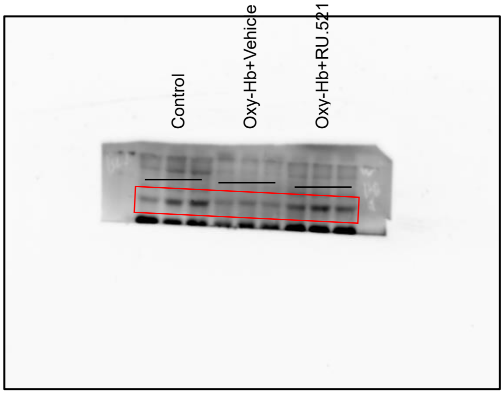

β-actin 42KD

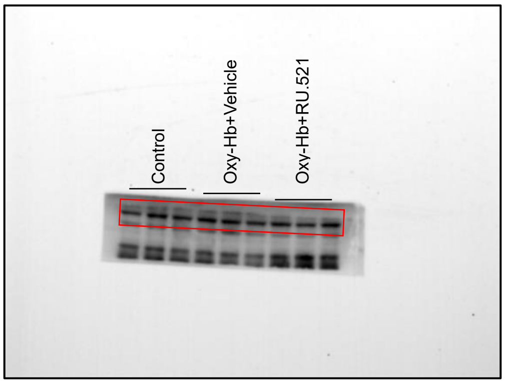

cytoplasm

NF-κB p65 65KD

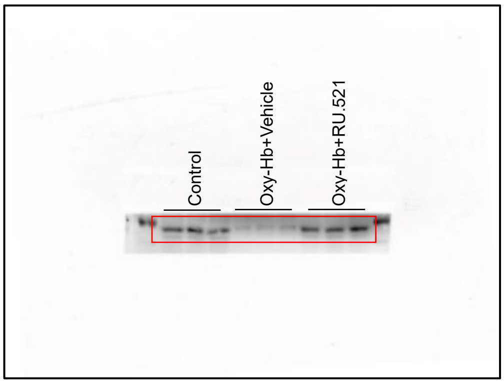

β-actin 42KD

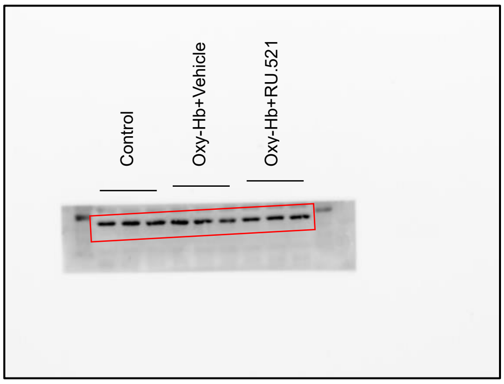

Lamin B1 66KD

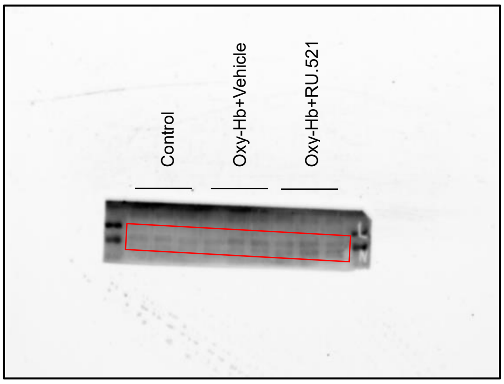

nuclear

NF-κB p65 65KD

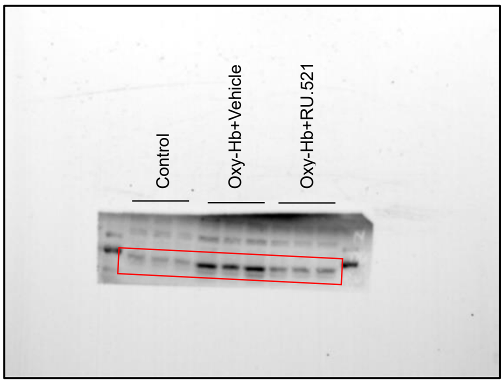

β-actin 42KD

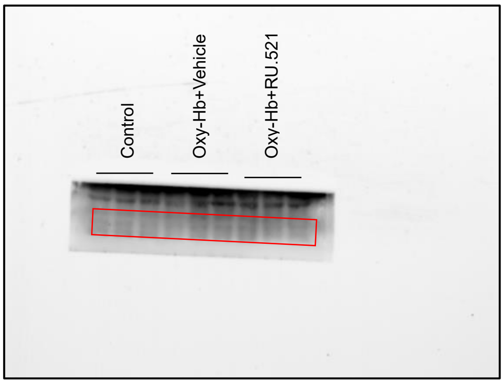

Lamin B1 66KD

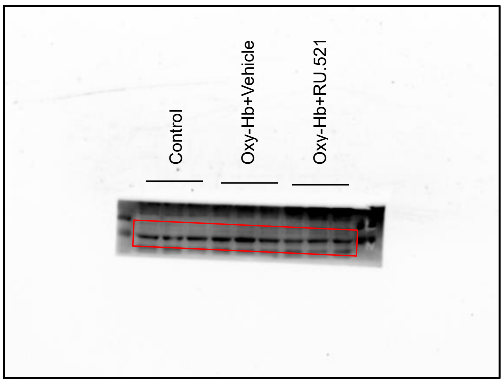

Supplement: Supplementary file 1 — Supplementary Material 2: Uncropped Western bot gel images. [file 12964_2024_1772_MOESM1_ESM.pdf]
